# New Biscembranoids Sardigitolides A–D and Known Cembranoid-Related Compounds from *Sarcophyton digitatum*: Isolation, Structure Elucidation, and Bioactivities

**DOI:** 10.3390/md18090452

**Published:** 2020-08-29

**Authors:** Tzu-Yin Huang, Chiung-Yao Huang, Chih-Hua Chao, Chi-Chien Lin, Chang-Feng Dai, Jui-Hsin Su, Ping-Jyun Sung, Shih-Hsiung Wu, Jyh-Horng Sheu

**Affiliations:** 1 Doctoral Degree Program in Marine Biotechnology, National Sun Yat-sen University, Kaohsiung 804, Taiwan; slime112229@gmail.com; 2 Department of Marine Biotechnology and Resources, National Sun Yat-sen University, Kaohsiung 804, Taiwan; huangcy@mail.nsysu.edu.tw; 3 School of Pharmacy, China Medical University, Taichung 404, Taiwan; chchao@mail.cmu.edu.tw; 4 Chinese Medicine Research and Development Center, China Medical University Hospital, Taichung 404, Taiwan; 5 Institute of Biomedical Science, National Chung-Hsing University, Taichung 402, Taiwan; lincc@dragon.nchu.edu.tw; 6 Department of Medical Research, China Medical University Hospital, China Medical University, Taichung 404, Taiwan; 7 Institute of Oceanography, National Taiwan University, Taipei 112, Taiwan; corallab@ntu.edu.tw; 8 National Museum of Marine Biology and Aquarium, Pingtung 944, Taiwan; x2219@nmmba.gov.tw (J.-H.S.); pjsung@nmmba.gov.tw (P.-J.S.); 9 Institute of Biological Chemistry and Chemical Biology and Molecular Biophysics Program, Taiwan International Graduate Program, Academia Sinica, Taipei 112, Taiwan; shwu@gate.sinica.edu.tw; 10 Graduate Institute of Natural Products, Kaohsiung Medical University, Kaohsiung 807, Taiwan

**Keywords:** *Sarcophyton digitatum*, biscembranoid-type metabolites, cytotoxicity, inflammatory factor production, LPS-stimulated murine macrophage

## Abstract

Chemical examination from the cultured soft coral *Sarcophyton digitatum* resulted in the isolation and structural identification of four new biscembranoidal metabolites, sardigitolides A–D (**1**–**4**), along with three previously isolated biscembranoids, sarcophytolide L (**5**), glaucumolide A (**6**), glaucumolide B (**7**), and two known cembranoids (**8** and **9**). The chemical structures of all isolates were elucidated on the basis of 1D and 2D NMR spectroscopic analyses. Additionally, in order to discover bioactivity of marine natural products, **1**–**8** were examined in terms of their inhibitory potential against the upregulation of inflammatory factor production in lipopolysaccharide (LPS)-stimulated murine macrophage J774A.1 cells and their cytotoxicities against a limited panel of cancer cells. The anti-inflammatory results showed that at a concentration of 10 µg/mL, **6** and **8** inhibited the production of IL-1*β* to 68 ± 1 and 56 ± 1%, respectively, in LPS-stimulated murine macrophages J774A.1. Furthermore, sardigitolide B (**2**) displayed cytotoxicities toward MCF-7 and MDA-MB-231 cancer cell lines with the IC_50_ values of 9.6 ± 3.0 and 14.8 ± 4.0 µg/mL, respectively.

## 1. Introduction

Cembrane diterpenoids and their derivatives are an abundant and structurally diverse group of secondary metabolites of soft corals and gorgonians. The 14-membered ring structures of cembranoids are biosynthetically formed by cyclization of a geranylgeranyl pyrophosphate precursor between carbons 1 and 14 [1]. These compounds possess a defense mechanism against their natural predators and protect corals against viral infections [2]. Cembranoids exhibit various useful biological properties, such as antitumor [3,4,5,6,7,8] and anti-inflammatory activities [7,9,10,11,12].

Previous studies indicated that species of *Sarcophyton* are rich sources of compounds, such as cembranoids [13,14,15,16], biscembranoids [17,18,19,20,21], and steroids [22]. Until now, more than 500 marine natural objects have been found from the soft corals of the genus *Sarcophyton* [23]. It is worth mentioning that biscembranoid-type metabolites are mainly biosynthetically rationalized by a Diels–Alder reaction of two monocembranoidal members, a 1,3-diene cembranoid unit, and a dienophile cembranoid unit. The diene unit forms new trisubstituted conjugated double bonds at C-34/C-35. Normally, most biscembranoids are biosynthesized by the endo-type cyclization of a Diels–Alder reaction [21]. Although many specimens of *Sarcophyton* genus have been chemically studied, there is only one chemical investigation of the soft coral *Sarcophyton digitatum* that has been reported by Zeng et al. in 2000, which led to the isolation of a polyhydroxylated sterol, sardisterol, from this species [24]. Due to the structure diversity and attracting bioactivities of natural products from soft corals, it is well worth further investigating for the discovery of new metabolites from *S. digitatum*. As part of our continuing investigations of discovering bioactive compounds from soft corals, herein is reported the chemical examination of a cultured *S. digitatum*, which resulted in the isolation and structure identification of four new biscembranoidal metabolites, sardigitolides A–D (**1**–**4**), along with five known compounds, sarcophytolide L (**5**) [25], glaucumolide A (**6**) [18], glaucumolide B (**7**) [18], isosarcophytonolide D (**8**) [26], and (4*Z*,8*S*,9*S*,12*Z*,14*E*)-9-hydroxy-1-isopropyl-8,12-dimethyloxabicyclo [9.3.2]-hexadeca-4,12,14-trien-18-one (**9**) [27]. Lipopolysaccharide (LPS), a known endotoxin, is the main component of the outer membrane of gram-negative bacteria, which could induce an inflammatory response in mammalian tissues. In most cases, the inflammatory response helps the organism to clear the LPS or pathogen. However, the violent inflammation caused by overproduced LPS could lead to tissue damage [28]. In the present study, the inhibitory effect of isolates **1**–**8** against the enhanced production of inflammatory factors in LPS-treated murine macrophage J774A.1 cells was examined. Furthermore, the in vitro cytotoxicities toward MCF-7, MDA-MB-231, HepG2, and HeLa cells of compounds **1**–**8** were investigated.

## 2. Results and Discussion

**Figure 1:**
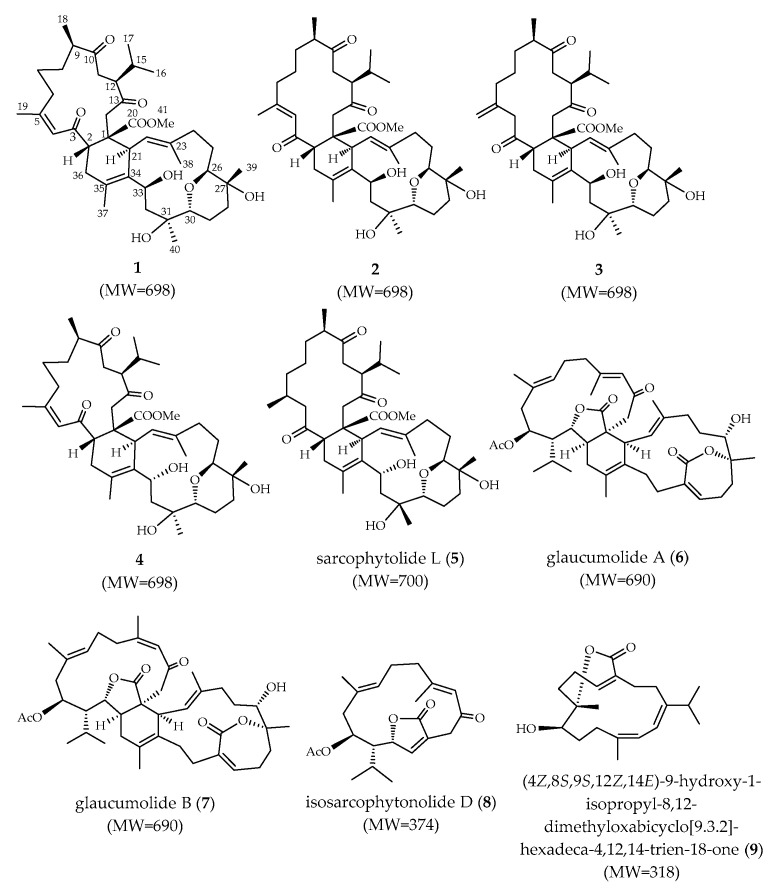
Structures of compounds **1**–**9**.

Soft coral *Sarcophyton digitatum* was cultured in the National Museum of Marine Biology & Aquarium. A frozen sample was initially extracted with *n*-hexane and subsequently by ethyl acetate. The ethyl acetate layer was concentrated and repeatedly chromatographed to isolate compounds **1**–**9** (Figure 1), and structure of new compounds **1**–**4** were established based on spectroscopic analyses (Supplementary Materials S1–S40).

**Figure 2:**
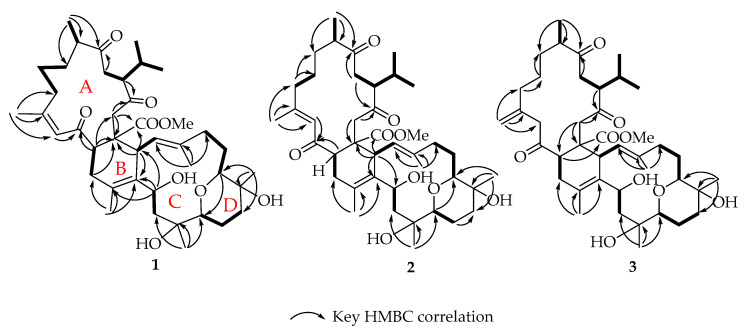
Selected COSY and HMBC correlations of **1**–**3**.

Sardigitolide A (**1**) was isolated as a white amorphous powder. High-resolution electron spray ionization mass spectroscopy (HRESIMS) showed a molecular ion peak at *m*/*z* 721.4287 ${\left[M\;+\;\mathrm{Na}\right]}^{+}$ corresponding to the molecular formula $C_{41}H_{62}O_{9}$ and implying 11 degrees of unsaturation. In the IR spectrum, a characteristic absorption at 3433 cm^−1^ indicated the presence of a hydroxy group. The ^13^C spectrum (Table 1) showed 41 carbon signals, including 9 methyl groups, 11 methylene, 10 methines, and 11 quaternary carbons. Its ^1^H (Table 2) and ^13^C NMR spectra revealed signals of three olefinic methyl groups (*δ*_H_ 1.89, s; 1.86, s; 1.70, s and *δ*_C_ 26.6; 19.3; 17.9, respectively), two methyl groups linked to oxygen-bearing quaternary carbons (*δ*_H_ 1.16, s; 1.13, s and *δ*_C_ 23.7; 25.3, respectively), one methoxy group (*δ*_H_ 3.55, s; *δ*_C_ 51.3), an isopropyl group (*δ*_H_ 2.19, m; 0.95, d, *J* = 6.8 Hz; 0.76, d, *J* = 6.8 Hz and *δ*_C_ 29.1, CH; 21.1, C$H_{3}$; 18.3, C$H_{3}$, respectively), two trisubstituted double bond (*δ*_H_ 6.44, s; *δ*_C_ 126.0, CH; 160.9, C and *δ*_H_ 5.06, d, *J* = 10.0 Hz; 125.2, CH; 138.4, C, respectively), one tetrasubstituted double bond (*δ*_C_ 131.7, C and 130.6, C), three oxygen-bearing methines (*δ*_H_ 4.86, t, *J* = 6.4 Hz; 3.49, m; 3.44, dd, *J* = 4.0, 12.0 Hz and *δ*_C_ 67.8, CH; 81.7, CH; 73.8, CH, respectively), two oxygen-bearing quaternary carbons (*δ*_C_ 73.8, C and 70.4, C), and four carbonyl carbons (*δ*_C_ 214.2, C; 210.0, C; 202.5, C; and 174.9, C). All of the above evidence suggested that **1** contained a biscembranoid skeleton. The planar structure of **1** was further determined. The correlations spectroscopy (COSY) signals revealed eight different structural units from H-2 to H_2_-36; H_2_-6 via H_2_-7 and H_2_-8; H-9 to H_3_-18; isopropyl protons H_3_-16 and H_3_-17 via H-15, H-12, and H-11; H-21 to H-22; H_2_-24 via H_2_-25 and H_2_-26; H_2_-28 via H_2_-29 and H-30; and H_2_-32 to H-33. These units were assembled by heteronuclear multiple bond correlations (HMBCs) of H_3_-16 to C-12, C-15, and C-17; H_3_-18 to C-8, C-9, and C-10; H_3_-19 to C-4, C-5, and C-6; H_3_-37 to C-34, C-35, and C-36; H_3_-38 to C-22, C-23, and C-24; H_3_-39 to C-27, C-28, and C-29; H_3_-40 to C-30, C-31, and C-32; H-2 to C-1, C-3, and C-14; H_2_-11 to C-10; H-12 to C-13; H_2_-14 to C-1 and C-20; and H-33 to C-34 and C-35. Accordingly, compound **1** had an additional degree of unsaturation, which was suggested to be of an ether ring between C-26 and C-30 established via an HMBC from H-26 to C-30. The gross structure of **1** was thus confirmed as shown in Figure 2 which possesses a biscembranoid skeleton similar to ximaolides E and F [29].

**Figure 3:**
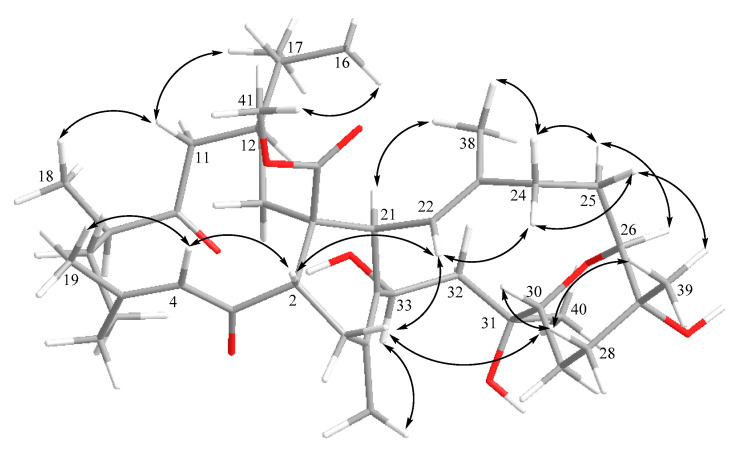
Key NOE correlations of compound **1**.

The relative configuration of **1** was determined by the analysis of correlations recorded by nuclear Overhauser effect spectroscopy (NOESY). As shown in Figure 3, the NOE correlations of H-4 (*δ*_H_ 6.44, s) with H_3_-19 (*δ*_H_ 1.89, s), together with the obviously downfield-shifted methyl group at C-19 (*δ*_C_ 26.6, CH_3_), suggested a *cis* geometry of C-4/C-5 trisubstituted double bond. Assuming the *β*-orientations of H_3_-41 as previously reported [30], NOE correlations of H_3_-41 with both of H_3_-16 (*δ*_H_ 0.76, d, *J* = 6.8 Hz) and H_3_-17 (*δ*_H_ 0.95, d, *J* = 6.8 Hz), and all of H_3_-16, H_3_-17 and H_3_-18 (*δ*_H_ 1.08, d, *J* = 7.2 Hz) with H-11 (*δ*_H_ 2.13, m) suggested the *β*-orientations of H_3_-18 and C-1 carbomethoxy groups, on the basis that metabolites of this type with absolute configurations determined previously all possessed 1*S* and 2*S* configurations [30]. Furthermore, the correlations of H-2 with H-22 (*δ*_H_ 5.06, d, *J* = 10.0 Hz); H-22 with H-24*β* (*δ*_H_ 2.43, m); and H-24*β* with H-25*β* (*δ*_H_ 1.84, m); H-25*β* with H_3_-39 (*δ*_H_ 1.13, s); H_3_-39 with H-28*β* (*δ*_H_ 1.52, m); H-28*β* with H-30 (*δ*_H_ 3.44, dd, *J* = 4.0, 12.0 Hz) reflected the *β*-orientation of H-30 and H_3_-39. In contrast, the *α*-orientations of H-9 and H-12 were confirmed by the NOE correlations of H-11*α* (*δ*_H_ 2.76, m) with both H-9 (*δ*_H_ 2.71, m) and H-12 (*δ*_H_ 2.97, m). The *α*-orientation of H-26 was determined by the NOE correlations of H-21 (*δ*_H_ 3.37, m) with H_3_-38 (*δ*_H_ 1.70, s); H_3_-38 with H-24*α* (*δ*_H_ 1.98, m); H-24*α* with H-25*α* (*δ*_H_ 1.64, m); H-25*α* with H-26 (*δ*_H_ 3.49, m). Also, the *α*-orientations of H-33 and H_3_-40 were determined by the NOE correlations of H-33 (*δ*_H_ 4.86, t, *J* = 6.4 Hz) with both H_3_-37 (*δ*_H_ 1.86, s) and H_3_-40 (*δ*_H_ 1.16, s). Moreover, the ^13^C NMR signals of C-37 (*δ*_C_ 19.3, CH_3_) and C-38 (*δ*_C_ 17.9, CH_3_) indicated the *E* geometry of the trisubstituted C-22/C-23 and tetrasubstituted C-34/C-35 double bonds. According to the above described evidence, and since compounds of this type are likely biosynthesized from the same or similar precursors and pathway, the absolute configuration of **1** was proposed as 1*S*,2*S*,9*R*,12*S*,21*S*,26*S*,27*R*,30*R*,31*S*,33*S*.

The pseudomolecular ion peak of sardigitolide B (**2**) ${\left[M\;+\;\mathrm{Na}\right]}^{+}$ at *m*/*z* = 721.4283 indicated the same molecular formula of **2**, C_41_H_62_O_9_, as that of **1**_._ The ^13^C NMR spectrum of **2** showed similar signals to those of **1**. Also, it displayed carbon signals of 9 methyls, 11 methylenes, 10 methines, and 11 quaternary carbons. By detailed analysis of the 2D NMR spectral data of **2**, a similar gross structure to that of **1** was deduced. However, it was found that **2** possesses a remarkably upfield-shifted signal of H-4 (*δ*_H_ 5.85, s), while **1** displayed a downfield-shifted resonance for H-4 (*δ*_H_ 6.44, s). Also, the ^13^C NMR spectroscopy data were found to be quite similar to those of **1**, with the exception of CH_3_-19 at *δ*_C_ 26.6 in **1** and *δ*_C_ 20.3 in **2**, suggesting the presence of a *trans* geometry of 4,5-double bond.

The relative configurations of the stereogenic centers in **2** were determined from the NOESY spectrum, which also showed similar NOE correlations to those of **1**. In contrast, H-4 (*δ*_H_ 5.85, s) showed an NOE correlation with H-2 (*δ*_H_ 3.73, m), but not with H_3_-19 (*δ*_H_ 2.12, s), confirming the *E* geometry of the double bond at C-4 and C-5 again. These results determined **2** to be the 4*E* isomer of **1**.

Sardigitolide C (**3**) exhibited a protonated molecular peak at *m*/*z* 721.4281 ${\left[M\;+\;\mathrm{Na}\right]}^{+}$ in the HR-ESI-MS spectrum, establishing the same molecular formula, C_41_H_62_O_9_, and 11 degrees of unsaturation as those of **1** and **2**. The ^13^C NMR spectroscopy data also showed 41 carbon signals, including 8 methyl groups, 13 methylene, 9 methines, and 11 quaternary carbons. Analyzing the 2D NMR spectral data of **3** in detail showed a similar gross structure of **3** to that of **1**, except that the chemical shifts of CH_2_-19 (*δ*_H_ 4.72, 4.88, brs; *δ*_C_ 114.9) in **3** indicated the presence of 1,1-disubstituted double bond at C-5/C-19. The relative configuration of **3** was also determined from NOESY spectrum, which showed similar NOE correlations among the corresponding protons to those of **1**. This is the first ximaolide-type compound containing a disubstituted double bond at C-5.

Sardigitolide D (**4**) was isolated as a white amorphous solid. The HR-ESI-MS of **4** showed a pseudomolecular ion peak at 721.4268 ${\left[M\;+\;\mathrm{Na}\right]}^{+}$, indicating the molecular formula C_41_H_62_O_9_ and implying 11 degrees of unsaturation. In the ^13^C and DEPT-135 spectra, **4** showed 41 carbon signals resembling those of **1** including 9 methyls, 11 methylenes, 10 methines, and 11 quaternary carbons. Also, **4** had the same gross structure as **1** according to the detailed analyses of 1D and 2D NMR (COSY and HMBC) spectra. However, it was found that H-33 of **4** showed a different coupling constant (*J* = 10.5 Hz) to that of H-33 (*J* = 6.4 Hz) in **1**. Furthermore, **4** did not show NOE correlation between H-22 (*δ*_H_ 4.99, d, *J* = 10.5 Hz) and H-33 (*δ*_H_ 4.78, d, *J* = 10.5 Hz), while **1**–**3** exhibited NOE interactions between H-22 and H-33, revealing the 33*R* configuration. On the basis of the above findings, the molecular structure of **4** was determined unambiguously to be the 33-epimer of **1**.

It is known that IL-1*β*, the cytokine family member of interleukin-1 produced by activated macrophages, can lead to a strong immune response, and many studies have shown that IL-1*β* is related to various diseases, such as rheumatoid arthritis [31,32], neuropathic pain [33], and cardiovascular disease [34]. Furthermore, IL-1*β* has been suggested as a therapeutic target for the treatment of diabetic retinopathy [35,36]. In this study, we also attempted to screen the bioactivity of isolates with potential anti-inflammatory and cytotoxic abilities. Therefore, **1**–**8** were screened for their inhibitory potential against the upregulation of proinflammatory cytokine IL-1*β* in LPS-stimulated murine macrophage J774A.1 cells. The results showed that, at a concentration of 10 *μ*g/mL, **6** and **8** potently inhibited LPS-induced IL-1*β* production to 68 ± 1 and 56 ± 1%, respectively, with IC_50_ values of 10.7 ± 2.7 and 14.9 ± 5.1 *μ*g/mL, respectively. In addition, compounds **1**–**8** were assayed for their cytotoxicities toward MCF-7, MDA-MB-231, HepG2, and HeLa carcinoma cell lines. The results (Table 3) showed that sardigitolide B (**2**) exhibited inhibitory activity toward MCF-7and MDA-MB-231 cells with IC_50_ values of 9.6 ± 3.0 and 14.8 ± 4.0 *μ*g/mL, respectively, and glacumolide A (**6**) showed cytotoxicity toward MCF-7, HepG2, and HeLa cell lines with IC_50_ values of 10.1 ± 3.3, 14.9 ± 3.5, and 17.1 ± 4.5 µg/mL, respectively. Glacumolide B (**7**) also was found to display cytotoxicity toward MCF-7, MDA-MB-231, and HepG2 cell lines, with IC_50_ values of 9.4 ± 3.0, 17.8 ± 4.5, and 14.9 ± 4.2 µg/mL, respectively. The monocembranoid **8** (molecular weight 374) showed cytotoxicity against the growth of MCF-7 cancer cell line with IC_50_ value of 10.9 ± 4.3 µg/mL (29.1 ± 11.4 *µ*M). The remaining compounds did not show any effective cytotoxic and anti-inflammatory activities.

**Figure 4:**
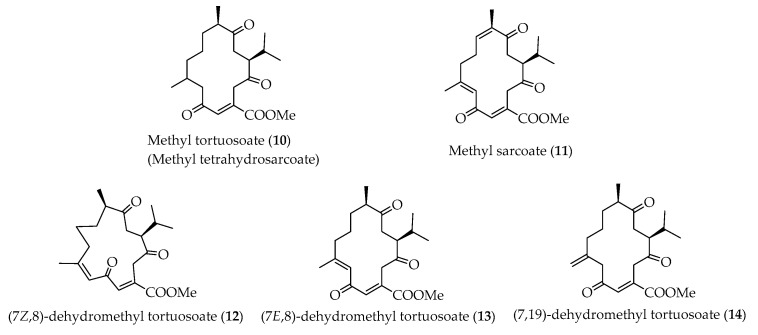
Structures of dienophiles **10**–**14**.

Methyl tortuosoate, also called methyl tetrahydrosarcoate (**10**) (Figure 4), has been frequently discovered to be the dienophile monomeric cembranoid in the Diels–Alder reaction to biosynthesize relevant biscembranoid, while methyl sarcoate (**11**) has been found to be the dienophile part of this series biscembranoids for only one time [37]. Biscembranoids **1** and **4** are the Diels–Alder reaction products using (7*Z*,8)-dehydromethyl tortuosoate (**12**), **2** using (7*E*,8)-dehydromethyl tortuosoate (**13**), and **3** using (7,19)-dehydromethyl tortuosoate (**14**), respectively, as the dienophiles in the biscembranoids biosynthesis. Cembranoids **12**–**14** are waiting for discovery in future investigations.

Our previous investigation of secondary metabolites from the soft coral *Sarcophyton glacum* led to the discovery of two biscembranoid-type compounds, glaucumolide A (**6**) and glaucumolide B (**7**) [19]. The absolute configurations of **6**, **7**, and analogs have been confirmed in 2019 by Wen et al. from the X-ray single crystal diffraction of the same compound **6**, isolated from the soft coral *S. trocheliophorum* [20]. Recently, with the development of molecular phylogenetic analysis technology, McFadden et al. suggested that *S. glacum* contained more than seven genetically distinct clades which might be the reason for the continuous discovery of high chemical diversity of secondary metabolites of the soft coral *S. glacum* [38]. For the same reason, it is possible that the corals of *Sarcophyton* genus could generate similar structure biscembranoids as our present study.

## 3. Materials and Methods

### 3.1. General Experimental Procedures

Optical rotations and IR spectra were recorded on a JASCO P-1020 polarimeter and FR/IR-4100 infrared spectrophotometer (Jasco Corporation, Tokyo, Japan), respectively. NMR spectra were acquired on a Varian Unity Inova 500 FT-NMR (Varian Inc., Palo Alto, CA, USA) at 500 and 125 MHz for ^1^H and ^13^C, respectively; or on Varian 400 MR FT-NMR instrument at 400 MHz and 100 MHz for ^1^H and ^13^C, respectively. The data of low-resolution electron spray ionization mass spectroscopy (LRESIMS) were measured by Bruker APEX II mass spectrometer. The data of HRESIMS were obtained by Bruker Apex-Qe 9.4T mass spectrometer. Silica gel 60 and reverse-phased silica gel (RP-18; 230–400 mesh) were used for normal-phase and reverse-phased column chromatography. Precoated silica gel plates (Kieselgel 60 F254, 0.2 mm) (Merck, Darmstadt, Germany) were used for analytical TLC. High-performance liquid chromatography was performed on a Hitachi L-2455 HPLC apparatus with a Supelco C18 column (ODS-3, 5 *μ*m, 250 × 20 mm; Sciences Inc., Tokyo, Japan).

### 3.2. Soft Coral Material

The cultured soft coral *Sarcophyton* *digitatum* of this study was originally collected from the wild in an 80-ton cultivation tank (height 1.6 m) located in the National Museum of Marine Biology and Aquarium, Taiwan. The tank was designed to simulate the fringing reefs in southern Taiwan [39]. The bottom of the tank was covered by a 3–5 cm layer of coral sand, and artificial rocks were distributed on the sand. Sand-filtered seawater pumped directly from the adjacent reef coast and added to the tank at an exchange rate of 10% per day of the total volume. The tank was a semiclosed recirculating aquaculture system and did not require deliberate feeding [40]. The water temperatures were maintained between 24 and 27 °C via a heat-exchanger cooling system. The soft coral *S. digitatum* was harvested in the National Museum of Marine Biology & Aquarium, Pingtung, in 2014. The coral was identified by one of the authors of this study (C.-F.D.). The animal sample was stored at the Department of Marine Biotechnology and Marine Resources, National Sun Yat-Sen University.

### 3.3. Extraction and Isolation

A sample of *S. digitatum* (0.9 kg) was sliced and exhaustively extracted with ethyl acetate (1 L × 5), and the solvent-free residue was sequentially partitioned between water and *n*-hexane, ethyl acetate, and *n*-butanol to yield three samples from the above solvents. The resulted EtOAc extract (1.098 g) was purified over silica gel by column chromatography and eluted with acetone in *n*-hexane (10–100%, stepwise), and then with methanol in acetone (0–100%, stepwise) to yield 13 fractions. Fraction 2 was eluted with the solvent system of acetone–*n*-hexane (6:1) to obtain 4 subfractions (A–D). Subfraction 2-B was purified by RP-HPLC using MeOH/H_2_O (3:1) at a flow rate of 5 mL/min to afford **8** (2.1 mg). Fraction 3 was eluted with acetone–*n*-hexane (1:5) followed by isolation over a silica gel column to yield 5 subfractions (A–E). Subfraction 3-D was also purified by RP-HPLC with the solvent system of MeOH/H_2_O (4:1) at a flow rate of 5 mL/min to obtain **9** (3.4 mg). Fraction 10 was eluted with acetone–*n*-hexane (1:3) and rechromatographed over RP-18 gel to obtain 6 subfractions (A–F). Subfraction 10-F was exhaustively purified by RP-HPLC using MeOH/H_2_O (2:1) at a flow rate of 5 mL/min to yield **6** (20.2 mg) and **7** (13.5 mg). Fraction 11, eluting with acetone–*n*-hexane (1:2), was carefully isolated by a reverse-phase silica gel column to yield 6 subfractions (A−F). Subfraction 11-C was further purified by RP-HPLC using MeOH/H_2_O (2:1) at a flow rate of 5 mL/min to obtain **1** (8.6 mg), **2** (4.0 mg), **3** (2.5 mg), **4** (2.0 mg), and **5** (23.3 mg).

Compound **1:** white powder; ${\left[\alpha \right]}_{D}^{25}$ = +48 (*c* 1.3, CHCl_3_), IR (KBr) *v*_max_ 3433, 2958, 1705, 1603, 1436, 1373, 1205, 1135, 1061, 1029 cm^−1^, ^13^C and ^1^H NMR data, Table 1 and Table 2; ESIMS *m*/*z* 721 ${\left[M\;+\;\mathrm{Na}\right]}^{+}$; HRESIMS *m*/*z* 721.4287 ${\left[M\;+\;\mathrm{Na}\right]}^{+}$(calcd for C_41_H_62_O_9_Na, 721.4292)

Compound **2:** white powder; ${\left[\alpha \right]}_{D}^{25}$ = +88 (*c* 0.1, CHCl_3_), IR (KBr) *v*_max_ 3398, 2927, 1704, 1607, 1372, 1204, 1135, 1070 cm^−1^, ^13^C and ^1^H NMR data, Table 1 and Table 2; ESIMS *m*/*z* 721 ${\left[M\;+\;\mathrm{Na}\right]}^{+}$; HRESIMS *m*/*z* 721.4283 ${\left[M\;+\;\mathrm{Na}\right]}^{+}$(calcd for C_41_H_62_O_9_Na, 721.4292)

Compound **3:** white powder; ${\left[\alpha \right]}_{D}^{25}$ = +39 (*c* 0.8, CHCl_3_), IR (KBr) *v*_max_ 3445, 2959, 1703, 1435, 1372, 1206, 1061 cm^−1^, ^13^C and ^1^H NMR data, Table 1 and Table 2; ESIMS *m*/*z* 721 ${\left[M\;+\;\mathrm{Na}\right]}^{+}$; HRESIMS *m*/*z* 721.4281 ${\left[M\;+\;\mathrm{Na}\right]}^{+}$(calcd for C_41_H_62_O_9_Na, 721.4292)

Compound **4:** white powder; ${\left[\alpha \right]}_{D}^{25}$ = +76 (*c* 0.5, CHCl_3_), IR (KBr) *v*_max_ 3446, 2957, 2925, 1706, 1603, 1435, 1375, 1206, 1061 cm^−1^, ^13^C and ^1^H NMR data, Table 1 and Table 2; ESIMS *m*/*z* 721 ${\left[M\;+\;\mathrm{Na}\right]}^{+}$; HRESIMS *m*/*z* 721.4268 ${\left[M\;+\;\mathrm{Na}\right]}^{+}$(calcd for C_41_H_62_O_9_Na, 721.4292)

### 3.4. Cytotoxicity Assay

Cell lines were purchased from the American Type Culture Collection (Rockville, MD). Cytotoxicity assays were carried out using the MTT assays [41]. MCF-7, MDA-MB-231, and HepG2, and HeLa cell lines were cultured and then exposed to various concentrations of **1**–**8** in dimethyl sulfoxide (DMSO) for 72 h to screen cytotoxicity. Doxorubicin, the positive control of the MTT assay, showed cytotoxicity towards MCF-7, MDA-MB-231, HepG2, and HeLa cancer cell lines, with IC_50_ values of, 0.7 ± 0.1, 1.3 ± 0.2, 1.2 ± 0.4, and 0.4 ± 0.1 (µg/mL), respectively.

### 3.5. Anti-Inflammatory Assay

Murine macrophage cell line J774A.1 was purchased from the American Type Culture Collection (Rockville, MD). J774A.1 cells were cultured in 96-well plates at a concentration of 5 × 10^4^ cells/mL and allowed to grow overnight. The cell cultures were added to the DMSO as control or different concentrations of compounds **1**–**8**, followed by treatment with LPS 1 µg/mL for 24 h. Determination of the cytokine concentration was performed by ELISA according to the manufacturer’s protocol and previously reported method [42].

## 4. Conclusions

Investigation of *Sarcophyton digitatum* led to the purification of four new biscembranoids **1**–**4**, along with five known compounds **5**–**9**. With regards to biological activities, compounds **2**, **6**, and **7** displayed inhibitory activities against the proliferation of a limited panel of cancer cell lines in a cytotoxic assay. Furthermore, compounds **6** and **8** showed anti-inflammatory activity, which inhibited LPS-induced enhanced IL-1*β* production. The soft coral *Sarcophyton* sp. has been shown to produce various biscembranoid metabolites. Up to date, more than 60 biscembranoids biosynthesized via Diels–Alder reaction were isolated. Interestingly, these biscembranoids and their biosynthetic monocembranoid precursors are often found in *Sarcophyton* species [17,18,19,20,21,30,43,44,45,46]. The results of this study suggest that aquaculture of *Sarcophyton* species might produce structurally diversified bioactive compounds that could be beneficial for future drug development. Furthermore, the three dienophiles **12**–**14** are waiting for discovery as new cembranoids in future studies.

## Figures and Tables

**Table 1:** ^13^C NMR spectroscopic data of **1**–**4.**

Position	1 ^α^	2	3	4 ^b^
1	50.2 (C)	48.7 (C)	49.8 (C)	49.4 (C)
2	46.0 (CH) ^c^	47.3 (CH)	45.8 (CH)	44.0 (CH)
3	202.5 (C)	203.8 (C)	210.3 (C)	201.5 (C)
4	126.0 (CH)	126.0 (CH)	51.0 (CH_2_)	124.7 (CH)
5	160.9 (C)	159.8 (C)	143.2 (C)	159.4 (C)
6	32.3 (CH_2_)	39.8 (CH_2_)	36.5 (CH_2_)	34.0 (CH_2_)
7	24.7 (CH_2_)	24.1 (CH_2_)	25.7 (CH_2_)	25.2 (CH_2_)
8	31.0 (CH_2_)	31.1 (CH_2_)	33.8 (CH_2_)	33.6 (CH_2_)
9	44.6 (CH)	45.7 (CH)	47.7 (CH)	44.7 (CH)
10	214.2 (C)	213.3 (C)	213.6 (C)	213.6 (C)
11	34.7 (CH_2_)	35.1 (CH_2_)	34.2 (CH_2_)	37.1 (CH_2_)
12	52.6 (CH)	52.1 (CH)	51.1 (CH)	52.3 (CH)
13	210.0 (C)	211.2 (C)	211.6 (C)	207.6 (C)
14	46.6 (CH_2_)	47.7 (CH_2_)	46.8 (CH_2_)	45.2 (CH_2_)
15	29.1 (CH)	29.4 (CH)	29.2 (CH)	28.3 (CH)
16	18.3 (CH_3_)	17.9 (CH_3_)	18.4 (CH_3_)	17.3 (CH_3_)
17	21.1 (CH_3_)	20.8 (CH_3_)	21.0 (CH_3_)	21.0 (CH_3_)
18	17.2 (CH_3_)	16.3 (CH_3_)	17.6 (CH_3_)	18.3 (CH_3_)
19	26.6 (CH_3_)	20.3 (CH_3_)	114.9 (CH_2_)	26.4 (CH_3_)
20	174.9 (C)	174.4 (C)	175.0 (C)	174.1 (C)
21	42.9 (CH)	40.8 (CH)	42.5 (CH)	41.7 (CH)
22	125.2 (CH)	124.0 (CH)	124.5 (CH)	126.8 (CH)
23	138.4 (C)	140.2 (C)	139.3 (C)	134.9 (C)
24	36.7 (CH_2_)	38.5 (CH_2_)	37.6 (CH_2_)	34.9 (CH_2_)
25	25.8 (CH_2_)	26.8 (CH_2_)	26.3 (CH_2_)	24.4 (CH_2_)
26	81.7 (CH)	84.8 (CH)	82.7 (CH)	79.8 (CH)
27	70.4 (C)	70.1 (C)	70.4 (C)	69.0 (C)
28	33.6 (CH_2_)	32.0 (CH_2_)	33.2 (CH_2_)	33.6 (CH_2_)
29	19.5 (CH_2_)	19.8 (CH_2_)	19.9 (CH_2_)	19.2 (CH_2_)
30	73.8 (CH)	74.0 (CH)	73.9 (CH)	72.8 (CH)
31	73.8 (C)	74.0 (C)	73.9 (C)	72.8 (C)
32	40.2 (CH_2_)	41.2 (CH_2_)	40.6 (CH_2_)	39.8 (CH_2_)
33	67.8 (CH)	69.9 (CH)	68.5 (CH)	64.9 (CH)
34	131.7 (C)	131.5 (C)	132.8 (C)	132.7 (C)
35	130.6 (C)	130.2 (C)	130.1 (C)	125.2 (C)
36	34.0 (CH_2_)	34.0 (CH_2_)	34.2 (CH_2_)	32.9 (CH_2_)
37	19.3 (CH_3_)	19.8 (CH_3_)	19.6 (CH_3_)	16.8 (CH_3_)
38	17.9 (CH_3_)	18.9 (CH_3_)	18.5 (CH_3_)	17.2 (CH_3_)
39	25.3 (CH_3_)	25.4 (CH_3_)	25.4 (CH_3_)	26.2 (CH_3_)
40	23.7 (CH_3_)	22.9 (CH_3_)	23.4 (CH_3_)	24.7 (CH_3_)
41	51.3 (CH_3_)	51.6 (CH_3_)	51.3 (CH_3_)	50.6 (CH_3_)

**^α^** Spectroscopic data of **1**–**3** were recorded at 100 MHz in CDCl_3_. **^b^** Spectroscopic data of **4** was recorded at 125 MHz in DMSO- *d*_6_. **^c^** Attached protons were deduced by DEPT experiments.

**Table 2:** ^1^H NMR spectroscopic data of **1**–**4**.

Position	1 ^α^	2	3	4 ^b^
2	3.59, m	3.73, m	3.74, t (7.6) ^c^	3.37, m
4	6.44, s	5.85, s	3.20, m; 3.64 m	6.30, s
6	1.68, m; 3.30, m	1.99, m; 2.21, m	1.80, m; 2.16 m	2.11, m
7	1.34, m; 1.52, m	1.51, m; 1.60, m	1.27, m	1.10, m; 1.30, m
8	1.34, m; 1.64, m	1.49, m	1.52, m; 1.63 m	1.23, m; 1.54, m
9	2.71, m	2.24, m	2.43, m	2.73, m
11	2.13, m; 2.76, m	2.09, m; 2.98, m	2.04, m; 3.0, m	2.05, m; 3.01, m
12	2.97, m	2.82, m	3.05, m	2.99, m
14	2.84, m; 3.11, m	2.58, m; 3.23, m	3.10, m	2.58, m; 2.82, m
15	2.19, m	2.07, m	2.03, m	2.03, m
16	0.76, d (6.8)	0.77, d (6.8)	0.79, d (6.8)	0.59, d (7.0)
17	0.95, d (6.8)	0.94, d (6.8)	0.95, d (6.4)	0.90, d (6.5)
18	1.08, d (7.2)	1.13, d (6.8)	1.13, d (7.2)	0.96, (7.0)
19	1.89, s	2.12, s	4.72, 4.88, brs	1.84, s
21	3.37, m	3.73, d (10.8)	3.50, m	3.36, m
22	5.06, d (10.0)	5.25, d (10.8)	5.17, d (10.4)	4.99, d (10.5)
24	1.98, m; 2.43, m	1.94, m; 2.52, m	1.94, m; 2.50 m	1.82, m; 2.24, m
25	1.64, m; 1.84, m	1.61, m; 1.99, m	1.63, m; 1.94 m	1.58, m; 1.64, m
26	3.49, m	3.60, m	3.54, d (9.6)	3.28, d (10.5)
28	1.52, m; 1.72, m	1.52, m; 1.71, m	1.58, m; 1.72 m	1.62, m; 2.16, m
29	1.57, m; 1.70, m	1.70, m;	1.63, m; 1.71 m	1.29, m; 1.64, m
30	3.44, dd (4.0; 12.0)	3.63, m	3.52, m	3.15, dd (4.0; 12.0)
32	1.55, m; 2.19 m	1.71, m; 1.92, m	1.60, m; 2.03 m	2.19, m
33	4.86, t (6.4)	4.80, t (6.4)	4.83, t (6.4)	4.78, d (10.5)
36	2.26, m	2.26 m; 2.56 m	2.15, m; 2.40, m	1.92, m
37	1.86, s	1.91, s	1.89, s	1.66, s
38	1.70, s	1.77, s	1.75, s	1.57, s
39	1.13, s	1.13, s	1.14, s	0.96, s
40	1.16, s	1.14, s	1.15, s	1.01, s
41	3.55, s	3.58, s	3.58, s	3.40, s

**^α^** Spectroscopic data of **1**–**3** were recorded at 400 MHz in CDCl_3_. **^b^** Spectroscopic data of **4** was recorded at 500 MHz in DMSO-*d*_6_. **^c^** *J* values (in Hz) in parentheses.

**Table 3:** Cytotoxicities of compounds **1**–**8.** IC_50_ (µg/mL) values are the mean ± SEM (*n* = 3).

Compound	MCF-7	MDA-MB-231	HepG2	HeLa
**1**	– ^a^	–	–	–
**2**	9.6 ± 3.0	14.8 ± 4.0	–	–
**3**	–	–	–	–
**4**	–	–	–	–
**5**	–	–	–	–
**6**	10.1 ± 3.3	–	14.9 ± 3.5	17.1 ± 4.5
**7**	9.4 ± 3.0	17.8 ± 4.5	14.9 ± 4.2	–
**8**	10.9 ± 4.3	–	–	–
Doxorubicin	0.7 ± 0.1	1.3 ± 0.2	1.2 ± 0.4	0.4 ± 0.1

^a^—> 20 µg/mL.

## Supplementary Materials

The following are available online at https://www.mdpi.com/1660-3397/18/9/452/s1, IR, HRESIMS, ^1^H, ^13^C, distortionless enhancement by polarization transfer (DEPT), heteronuclear single quantum coherence spectroscopy (HSQC), COSY, HMBC, and NOESY spectra of new Compounds **1**–**4** are available online at https://www.mdpi.com/1660-3397/18/9/452/s1, Figure S1: IR spectrum of **1**, Figure S2: ESIMS spectrum of **1**, Figure S3: HRESIMS spectrum of **1**, Figure S4: ^1^H NMR spectrum of **1** in CDCl_3_ at 400 MHz, **Figure S5**: ^13^C NMR spectrum of **1** in CDCl_3_ at 100 MHz, Figure S6: DEPT spectrum of **1**, **Figure S7**: HSQC spectrum of **1**, **Figure S8**: COSY spectrum of **1**, Figure S9: HMBC spectrum of **1**, Figure S10: NOESY spectrum of **1**, **Figure S11**: IR spectrum of **2**, Figure S12: ESIMS spectrum of **2,** Figure S13: HRESIMS spectrum of **2**, Figure S14: ^1^H NMR spectrum of **2** in CDCl_3_ at 400 MHz, **Figure S15**: ^13^C NMR spectrum of **2** in CDCl_3_ at 100 MHz, Figure S16: DEPT spectrum of **2**, **Figure S17**: HSQC spectrum of **2**, **Figure S18**: COSY spectrum of **2**, Figure S19: HMBC spectrum of **2**, Figure S20: NOESY spectrum of **2**, Figure S21: IR spectrum of **3**, Figure S22: ESIMS spectrum of **3**, Figure S23: HRESIMS spectrum of **3**, Figure S24: ^1^H NMR spectrum of **3** in CDCl_3_ at 400 MHz, **Figure S25**: ^13^C NMR spectrum of **3** in CDCl_3_ at 100 MHz, **Figure S26**: DEPT spectrum of **2**, Figure S27: HSQC spectrum of **3**, **Figure S28**: COSY spectrum of **3**, Figure S29: HMBC spectrum of **3**, Figure S30: NOESY spectrum of **3**, Figure S31: IR spectrum of **4**, Figure S32. ESIMS spectrum of **3,** Figure S33: HRESIMS spectrum of **4**, Figure S34: ^1^H NMR spectrum of **4** in DMSO-*d*_6_ at 500 MHz, **Figure S35**: ^13^C NMR spectrum of **4** in DMSO-*d*_6_ at 125 MHz, Figure S36: DEPT spectrum of **4****, Figure S37**: HSQC spectrum of **4**, Figure S38: COSY spectrum of **4**, Figure S39: HMBC spectrum of **4**, Figure S40: NOESY spectrum of **4**. Figure S41: ESIMS spectrum of **5**, **Figure S42**: ^1^H NMR spectrum of **5** in CDCl_3_ at 400 MHz, Figure S43: ^13^C NMR spectrum of **5** in CDCl_3_ at 100 MHz, Figure S44: ESIMS spectrum of **6**, Figure S45: ^1^H NMR spectrum of **6** in CDCl_3_ at 400 MHz, Figure S46: ^13^C NMR spectrum of **6** in CDCl_3_ at 100 MHz, Figure S47: ESIMS spectrum of **7**, Figure S48: ^1^H NMR spectrum of **7** in CDCl_3_ at 400 MHz, Figure S49: ^13^C NMR spectrum of **7** in CDCl_3_ at 100 MHz, Figure S50: ESIMS spectrum of **8**, Figure S51: ^1^H NMR spectrum of **8** in CDCl_3_ at 500 MHz, Figure S52: ^13^C NMR spectrum of **8** in CDCl_3_ at 125 MHz.
