# Supplementary material for: New Biscembranoids Sardigitolides A–D and Known Cembranoid-Related Compounds from Sarcophyton digitatum: Isolation, Structure Elucidation, and Bioactivities
Source: Mar Drugs. 2020 Aug 29;18(9):452. doi: 10.3390/md18090452 (PMC7551163; doi:10.3390/md18090452)
Supplement: Supplementary file 1 [file marinedrugs-18-00452-s001.pdf]

# New Biscembranoids Sardigitolides A–D and Known Cembranoid-Related Compounds from *Sarcophyton digitatum*: Isolation, Structure Elucidation, and Bioactivities

**Tzu-Yin Huang [1], Chiung-Yao Huang [2], Chih-Hua Chao [3,4], Chi-Chien Lin [5,6], Chang-Feng Dai [7], Jui-Hsin Su [8], Ping-Jyun Sung [8], Shih-Hsiung Wu [9] and Jyh-Horng Sheu [1,2,6,10,*]**

[1] Doctoral Degree Program in Marine Biotechnology, National Sun Yat-sen University, Kaohsiung 804, Taiwan; slime112229@gmail.com
[2] Department of Marine Biotechnology and Resources, National Sun Yat-sen University, Kaohsiung 804, Taiwan; huangcy@mail.nsysu.edu.tw
[3] School of Pharmacy, China Medical University, Taichung 404, Taiwan; chchao@mail.cmu.edu.tw
[4] Chinese Medicine Research and Development Center, China Medical University Hospital, Taichung 404, Taiwan
[5] Institute of Biomedical Science, National Chung-Hsing University, Taichung 402, Taiwan; lincc@dragon.nchu.edu.tw
[6] Department of Medical Research, China Medical University Hospital, China Medical University, Taichung 404, Taiwan
[7] Institute of Oceanography, National Taiwan University, Taipei 112, Taiwan; corallab@ntu.edu.tw
[8] National Museum of Marine Biology and Aquarium, Pingtung 944, Taiwan; x2219@nmmba.gov.tw (J.-H.S.); pjsung@nmmba.gov.tw (P.-J.S.)
[9] Institute of Biological Chemistry and Chemical Biology and Molecular Biophysics Program, Taiwan International Graduate Program, Academia Sinica, Taipei, Taiwan; shwu@gate.sinica.edu.tw
[10] Graduate Institute of Natural Products, Kaohsiung Medical University, Kaohsiung 807, Taiwan
* Correspondence: sheu@mail.nsysu.edu.tw; Tel.: +886-7-5252000 (ext. 5030); Fax: +886-7-5255020

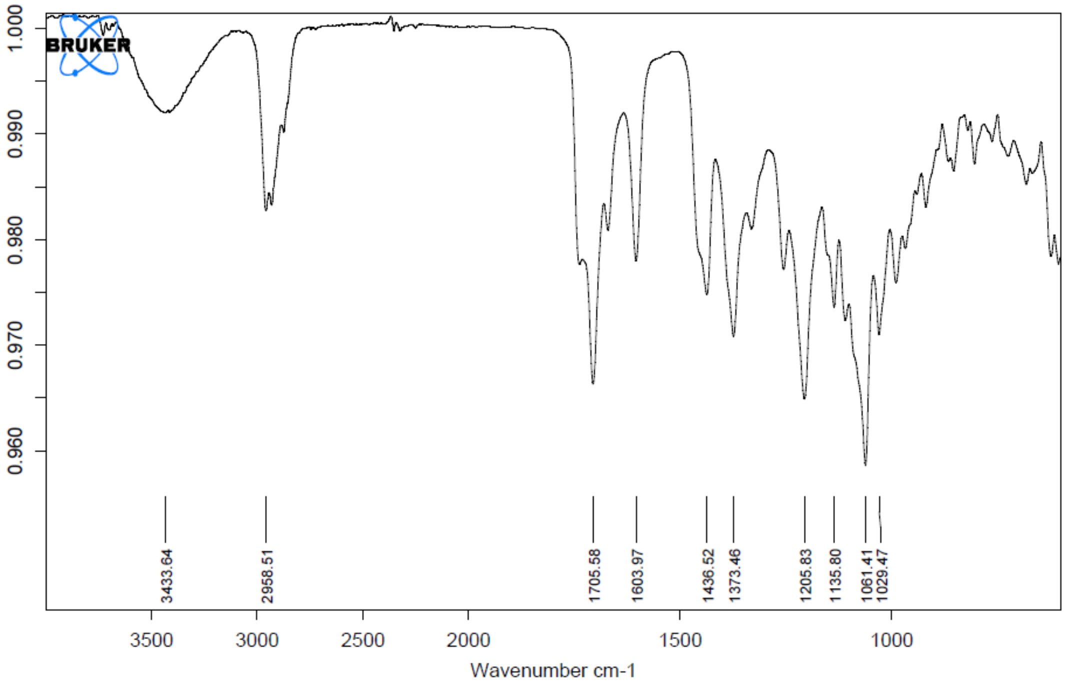


Figure S1: IR spectrum of **1**

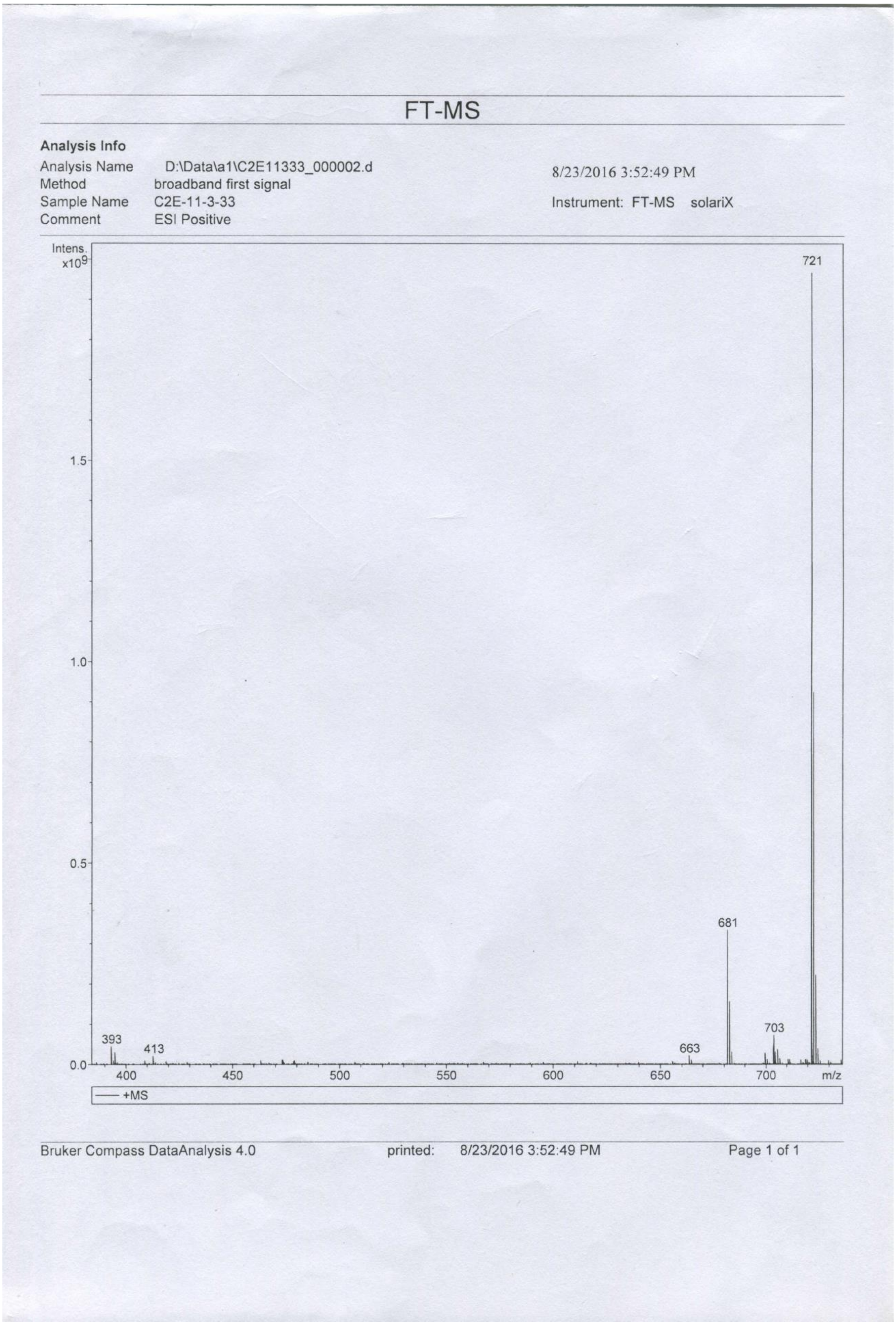
FT-MS

**Analysis Info**

Analysis Name D:\Data\a1\C2E11333_000002.d
Method broadband first signal
Sample Name C2E-11-3-33
Comment ESI Positive

8/23/2016 3:52:49 PM
Instrument: FT-MS solariX



Bruker Compass DataAnalysis 4.0 printed: 8/23/2016 3:52:49 PM Page 1 of 1

Figure S2: ESIMS spectrum of **1**

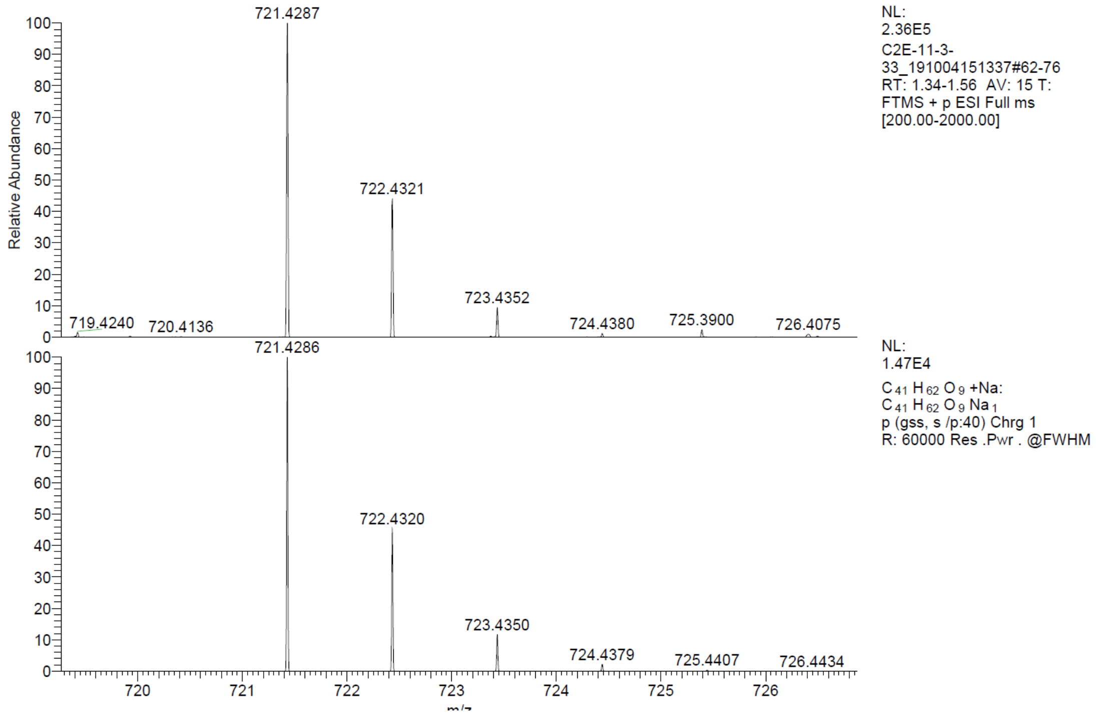


Figure S3: HRESIMS spectrum of **1**

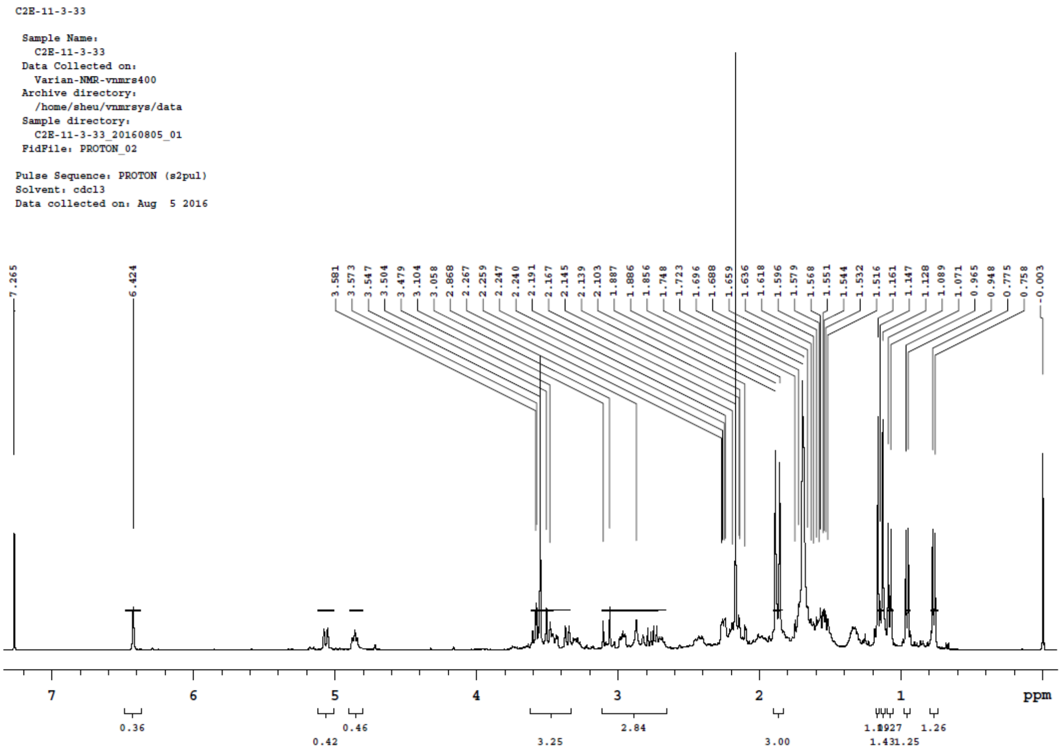


Figure S4: $^{1}$H NMR spectrum of **1** in $CDCl_3$ at 400 MHz

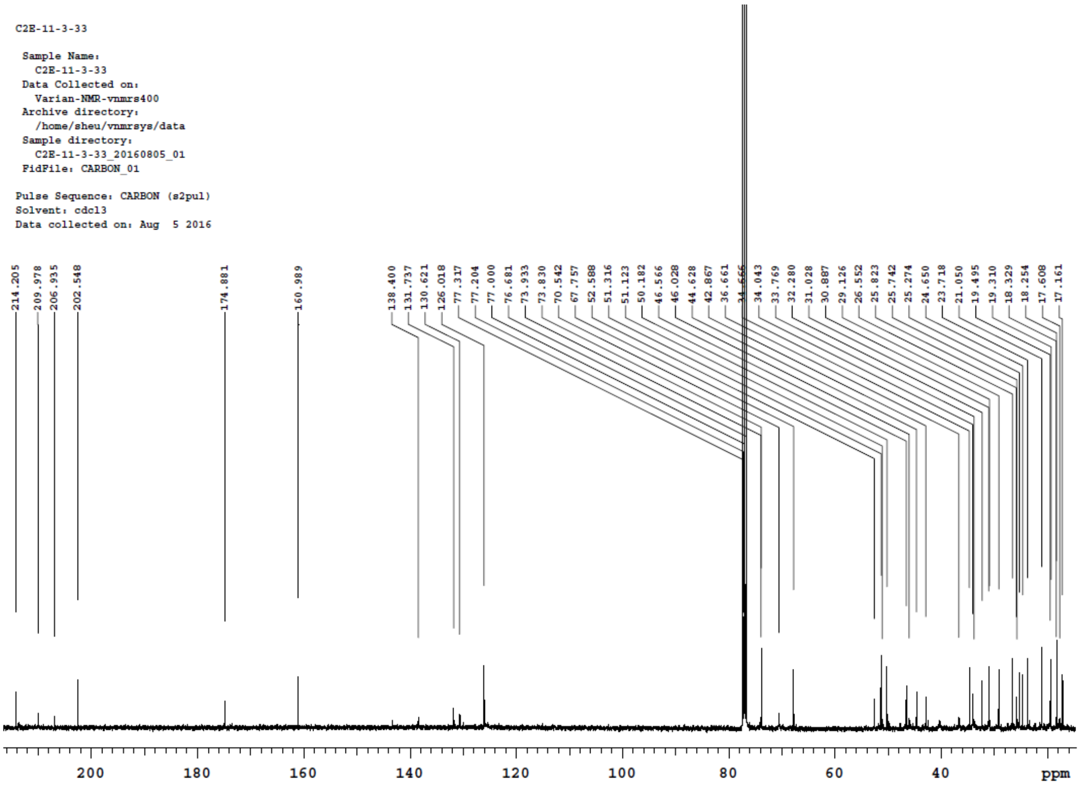


Figure S5: $^{13}$C NMR spectrum of **1** in $CDCl_3$ at 100 MHz

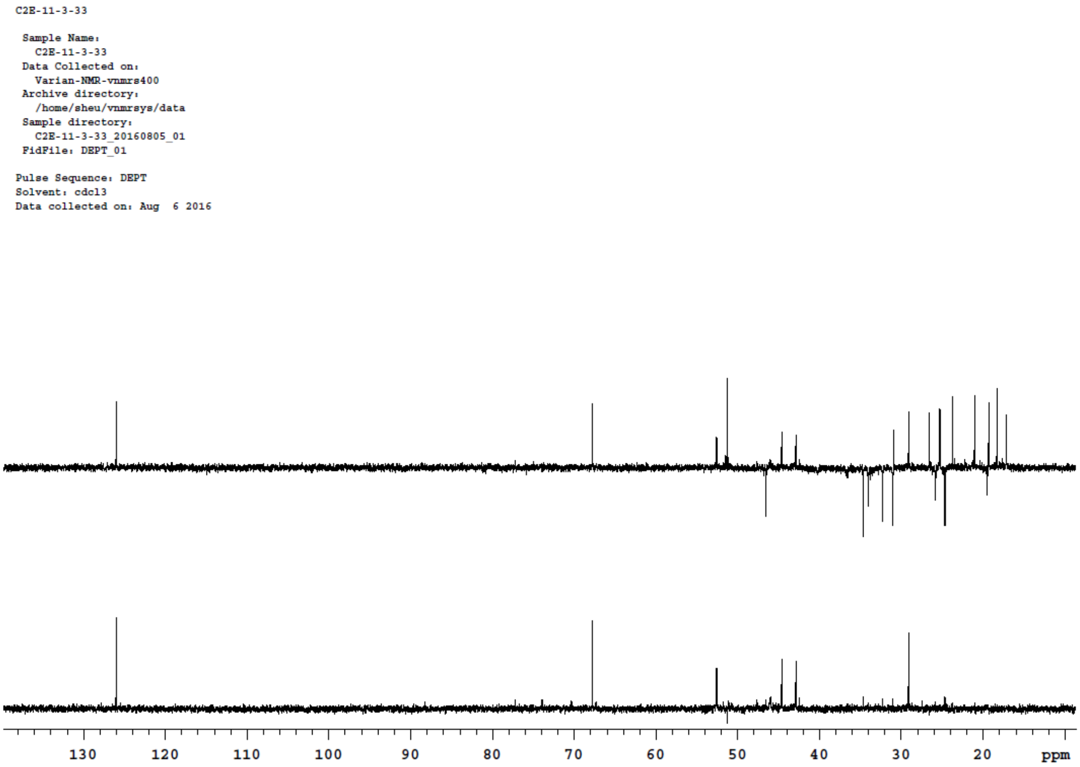


Figure S6: DEPT spectrum of **1**

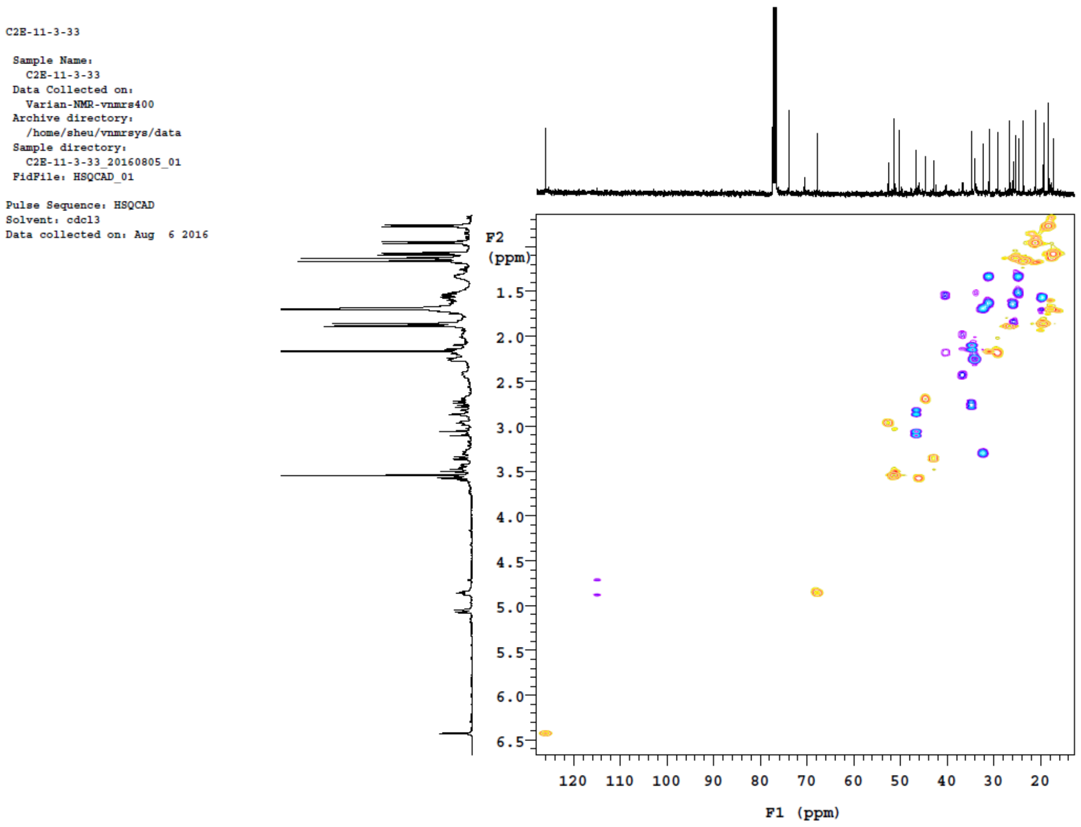


Figure S7: HSQC spectrum of **1**

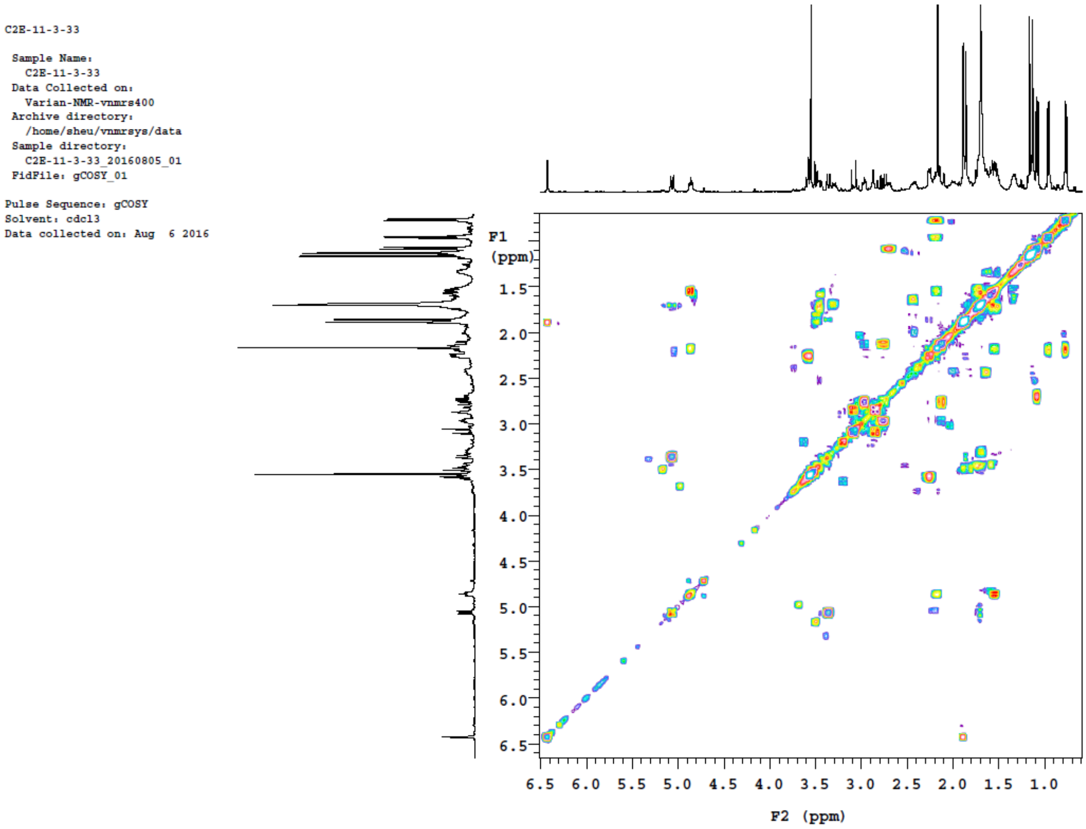


Figure S8: COSY spectrum of **1**

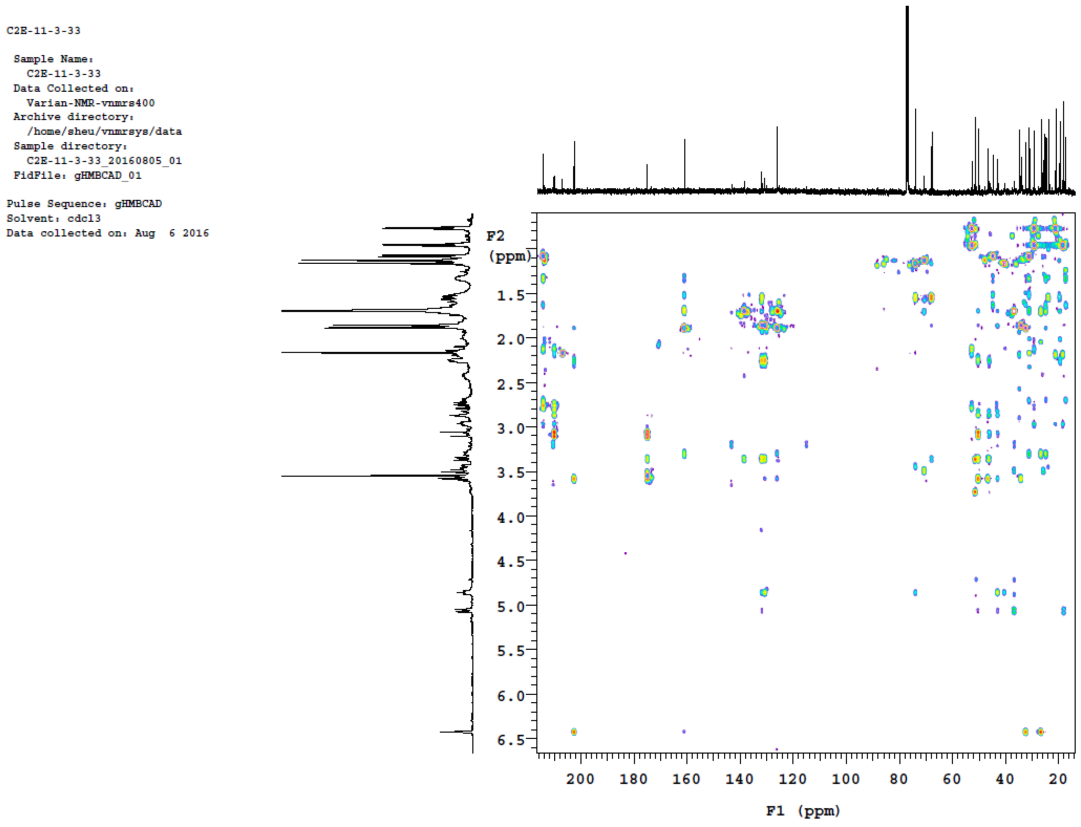


Figure S9: HMBC spectrum of **1**

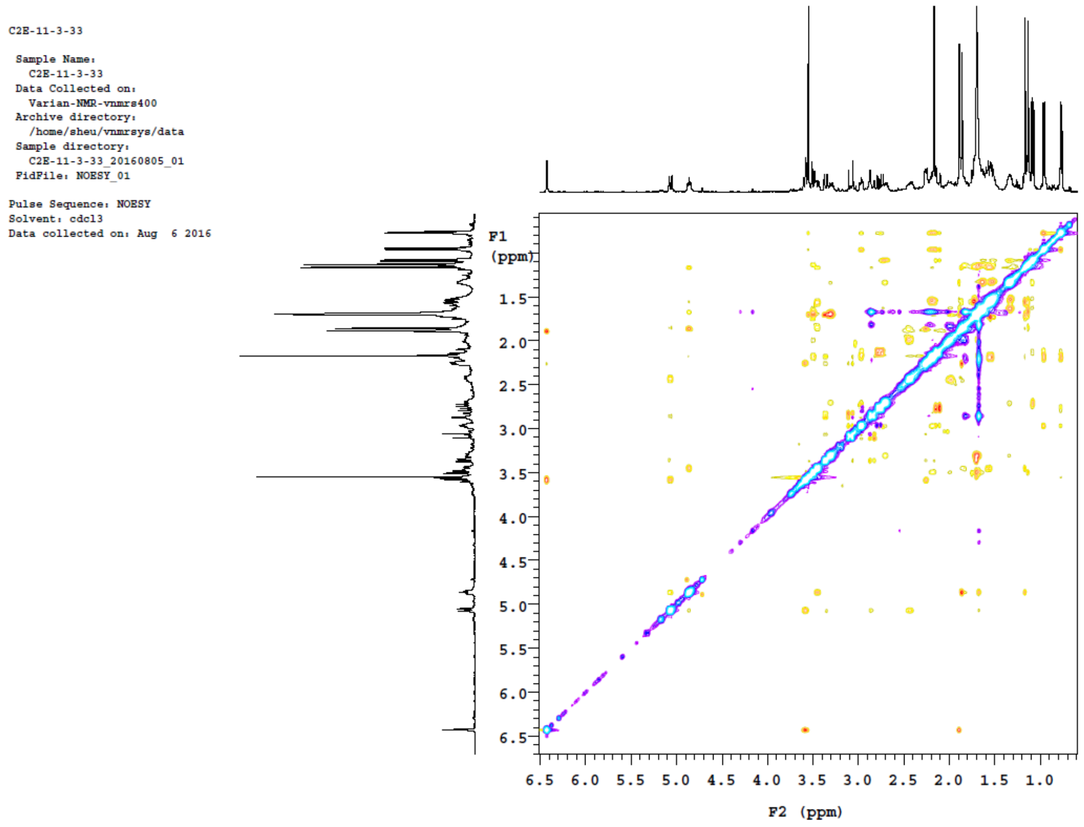


Figure S10: NOESY spectrum of **1**

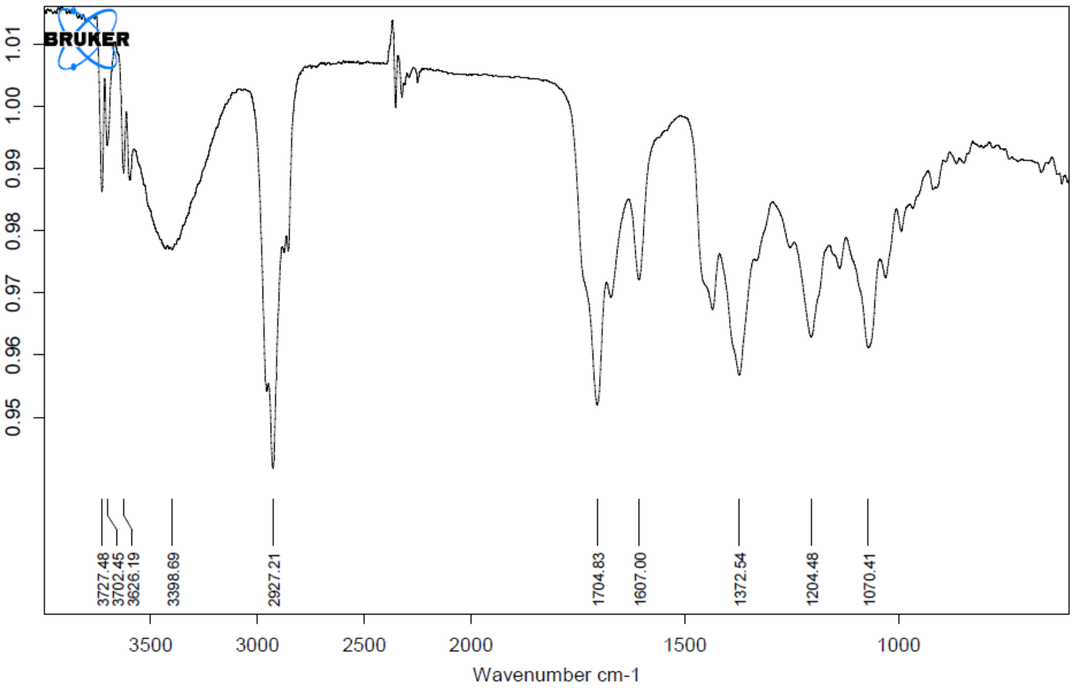


Figure S11: IR spectrum of **2**

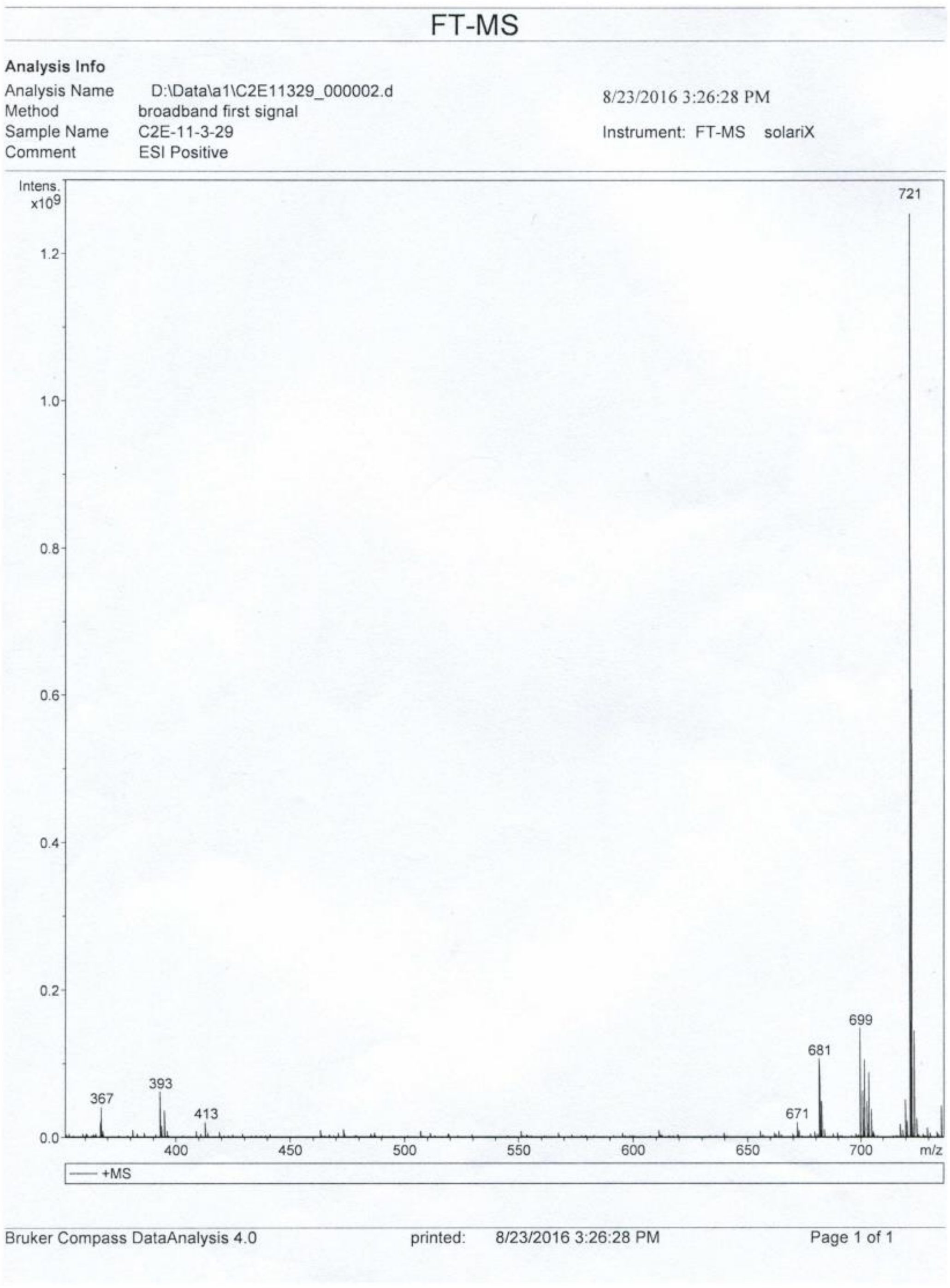


Figure 12: ESIMS spectrum of **2**

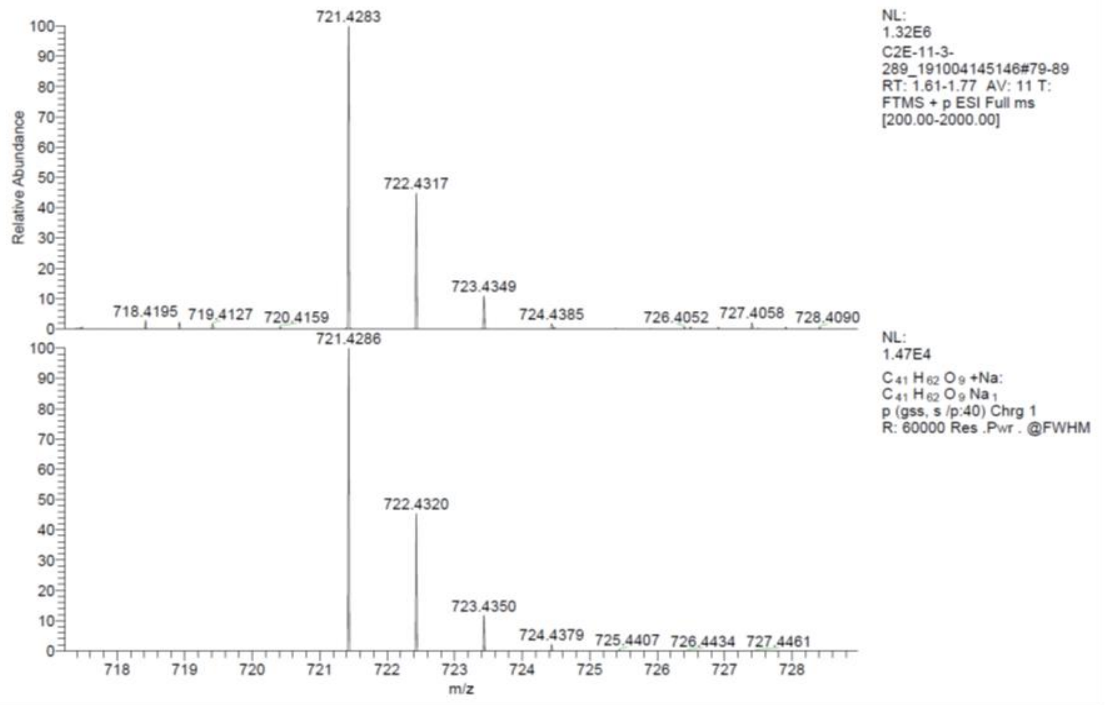


Figure S13: HRESIMS spectrum of **2**

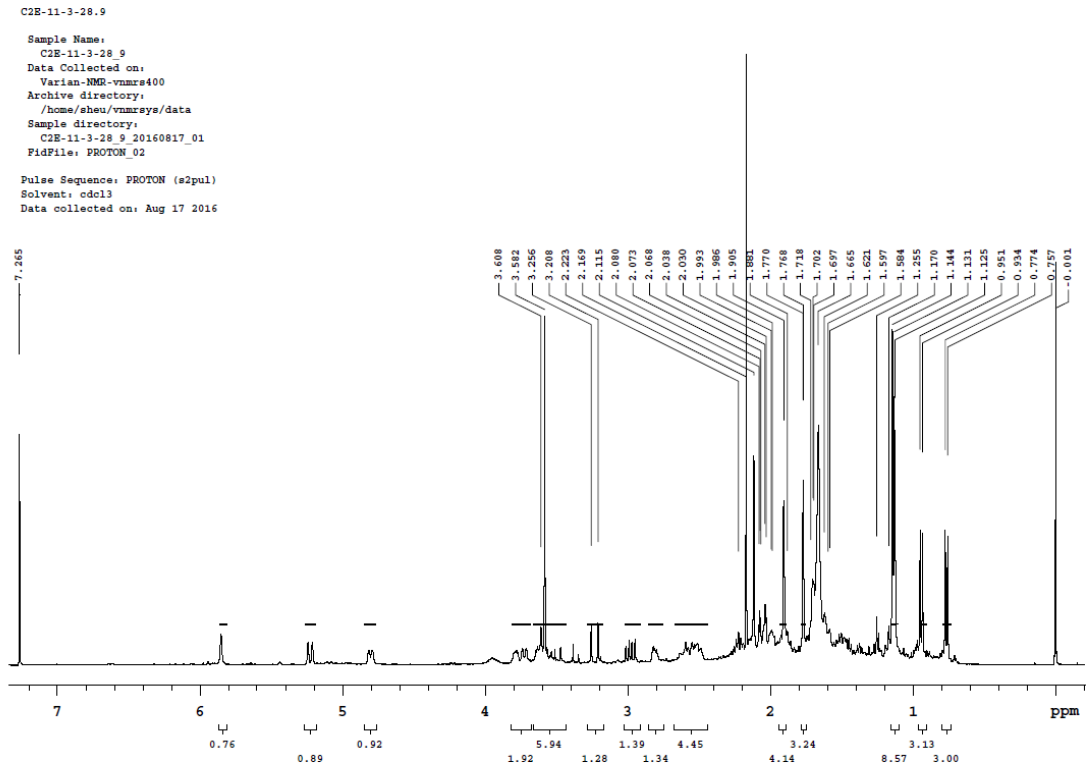


Figure S14: $^{1}$H NMR spectrum of **2** in $CDCl_3$ at 400 MHz

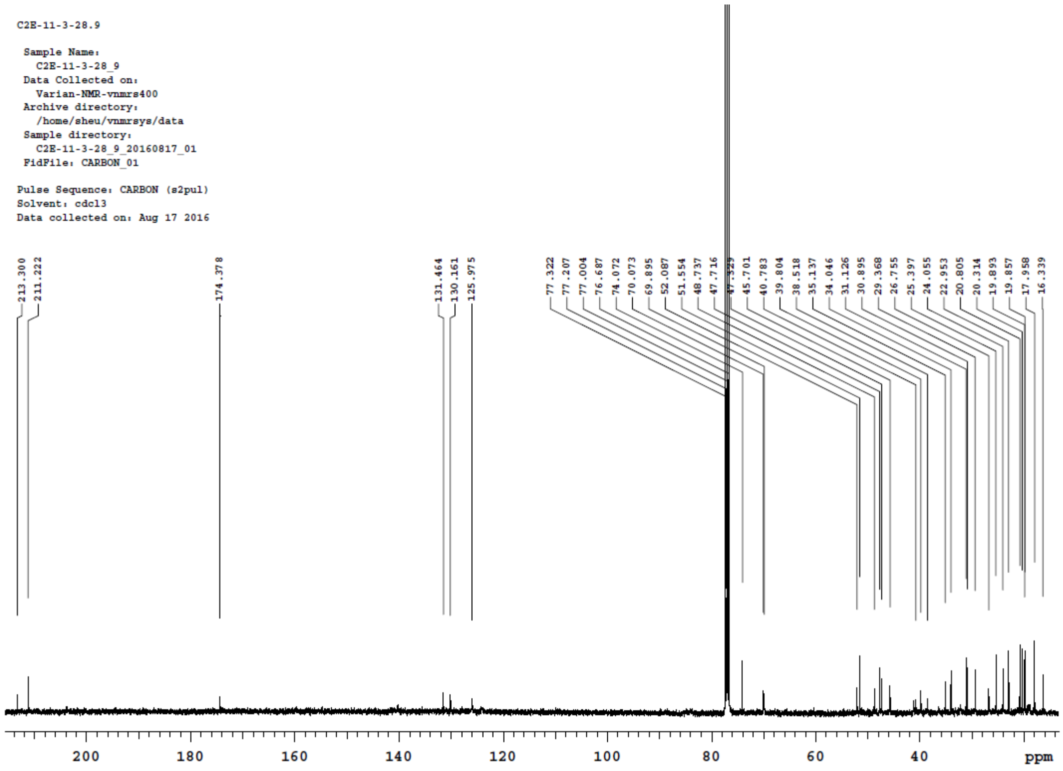


Figure S15: $^{13}$C NMR spectrum of **2** in $CDCl_3$ at 100 MHz

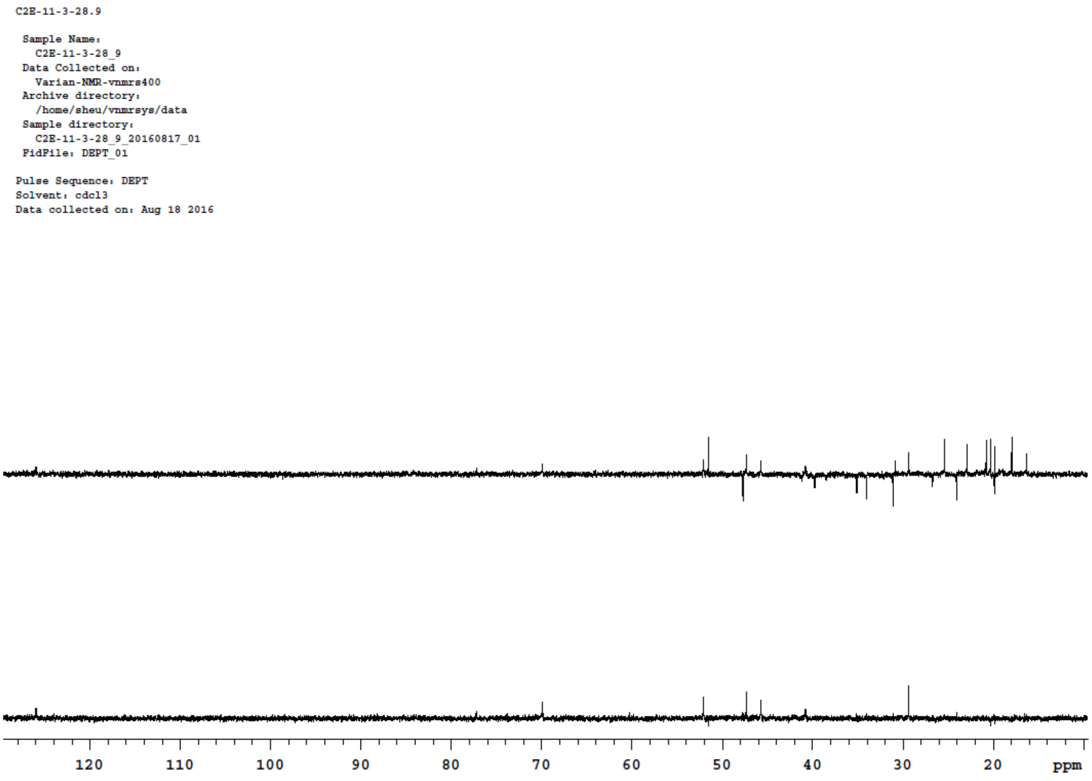


Figure S16: DEPT spectrum of **2**

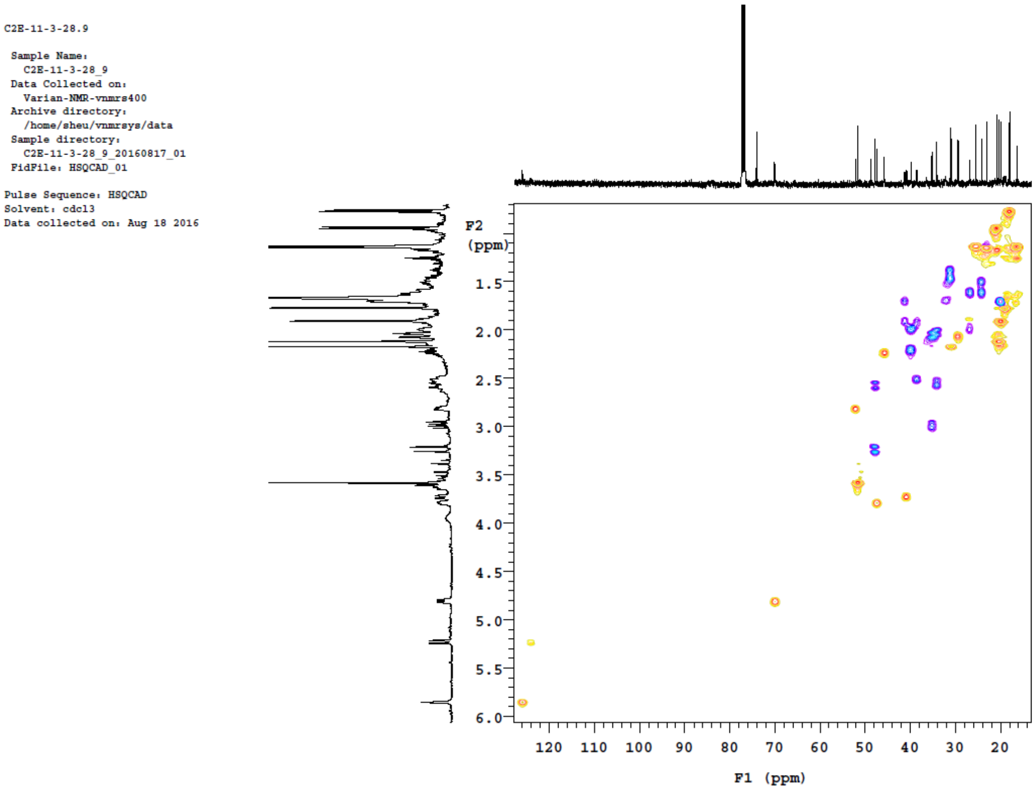


Figure S17: HSQC spectrum of **2**

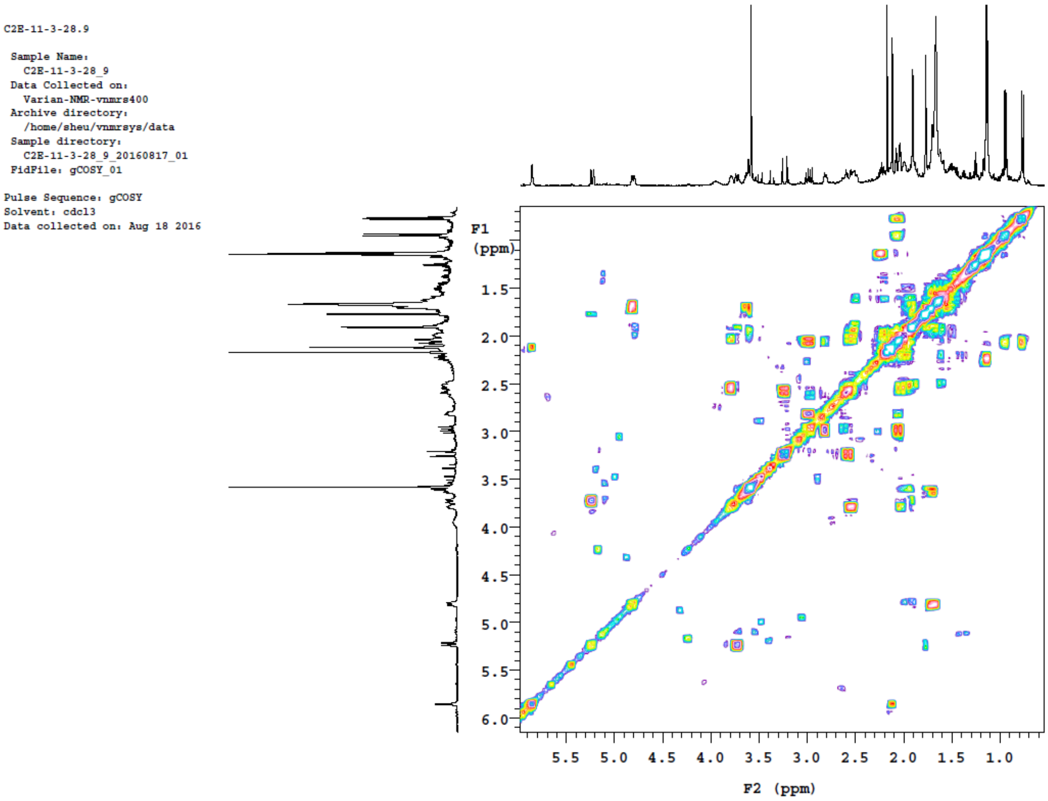


Figure S18: COSY spectrum of **2**

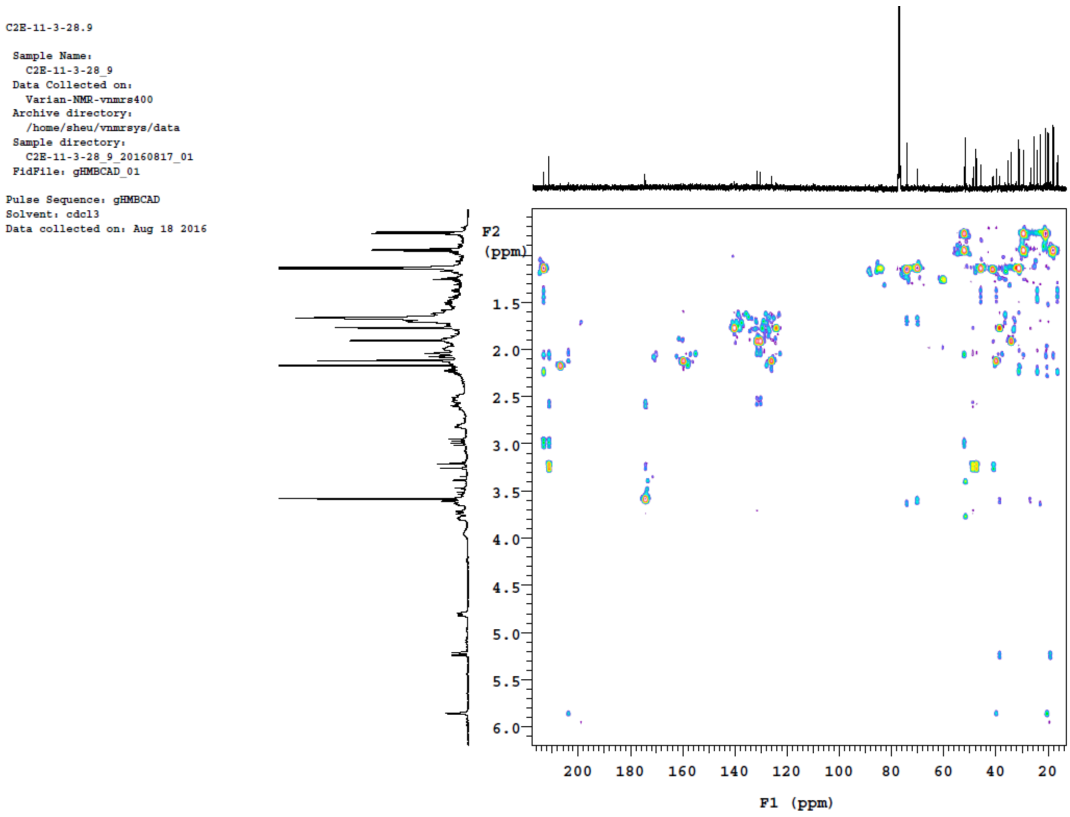


Figure S19: HMBC spectrum of **2**

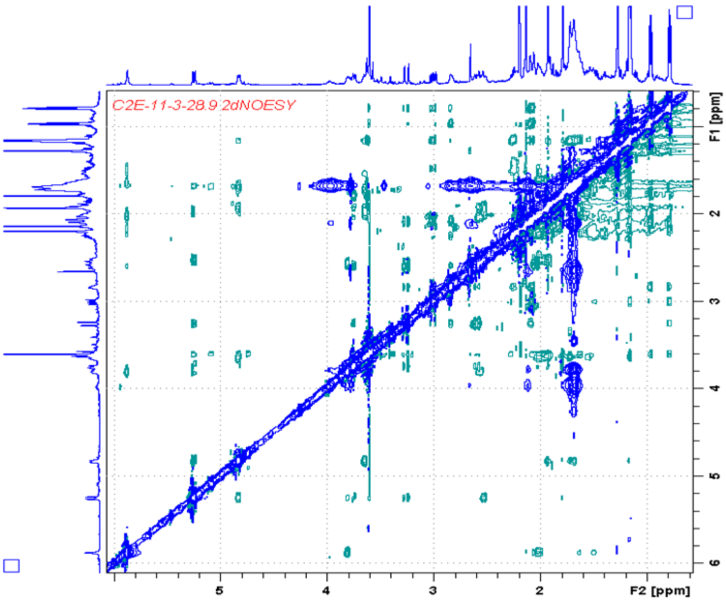


Figure S20: NOESY spectrum of **2**

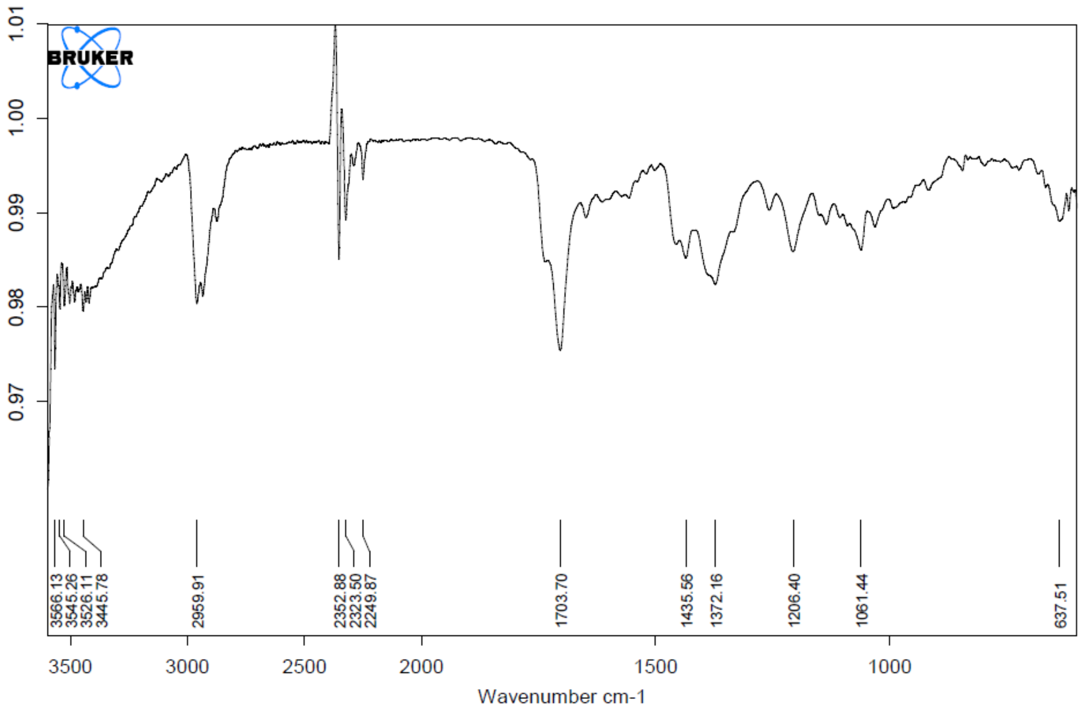

Figure S21: IR spectrum of **3**

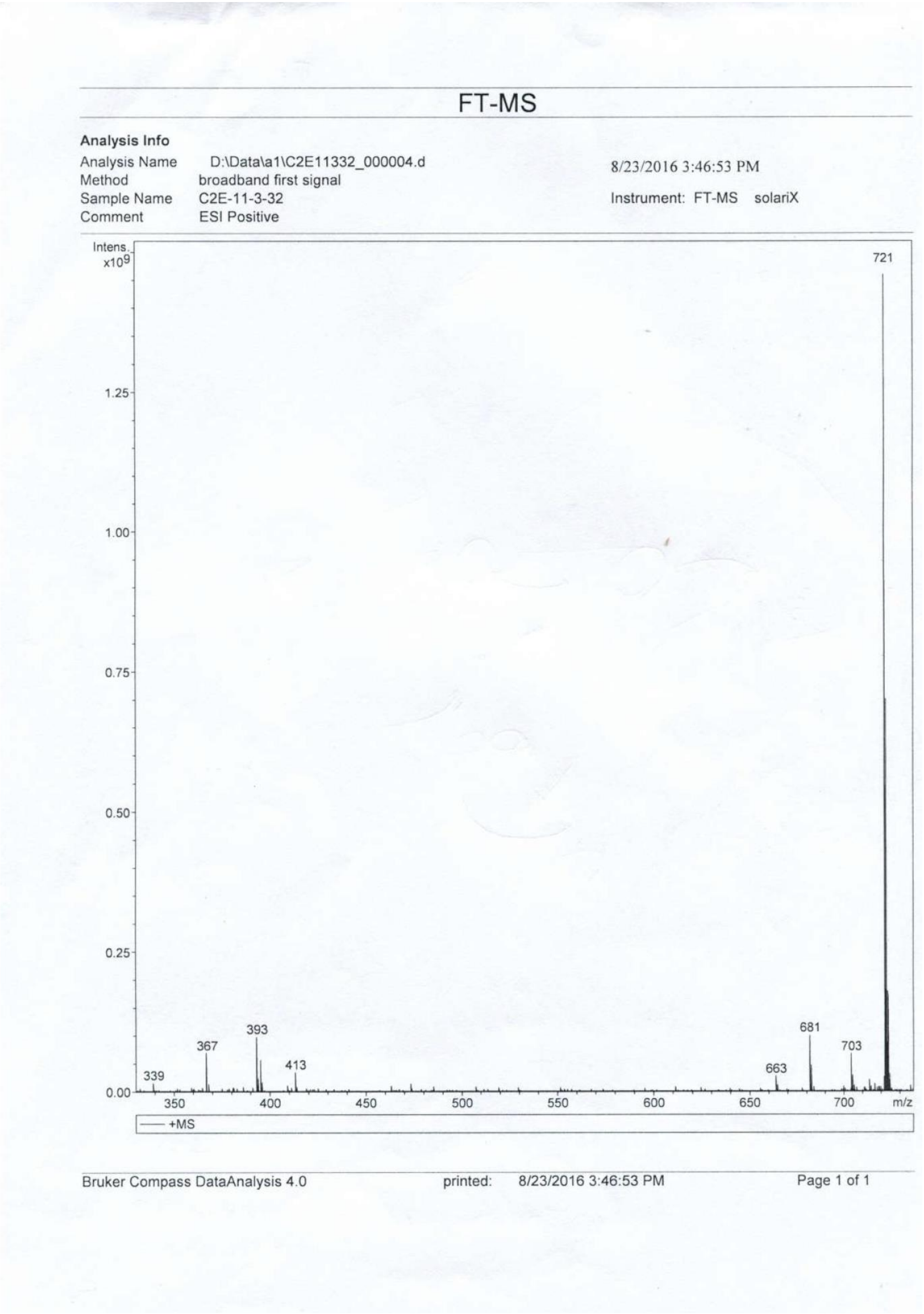


Figure 22: ESIMS spectrum of **3**

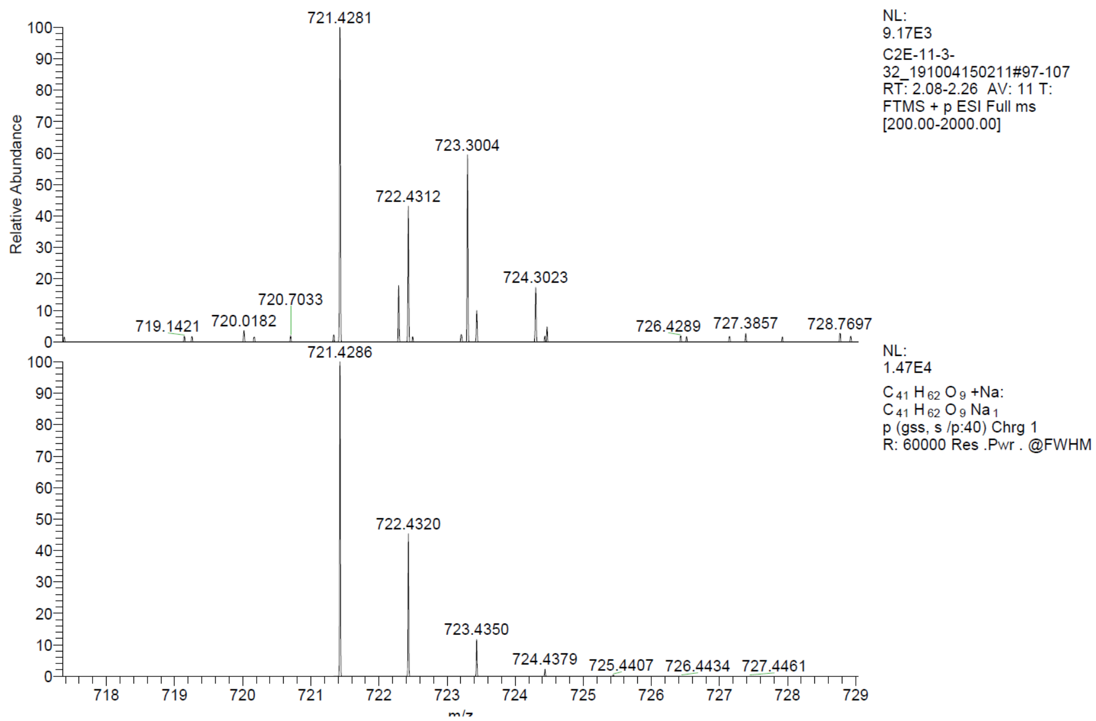


Figure S23: HRESIMS spectrum of **3**

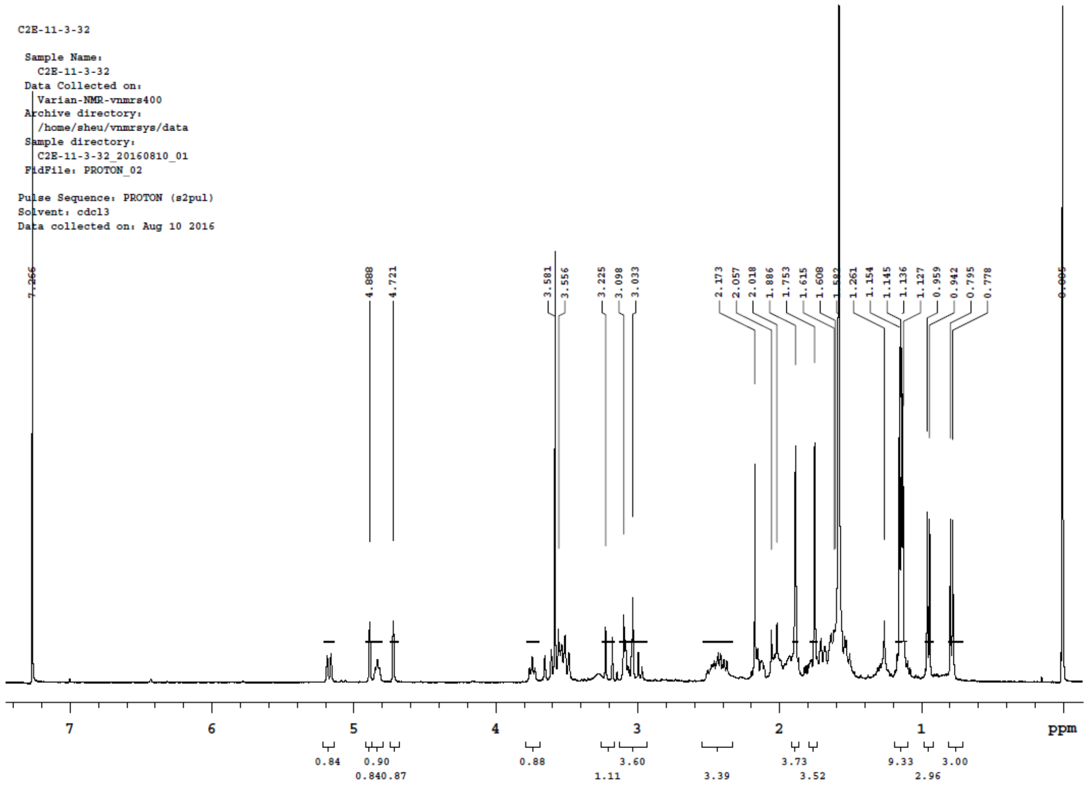


Figure S24: $^1$H NMR spectrum of **3** in $CDCl_3$ at 400 MHz

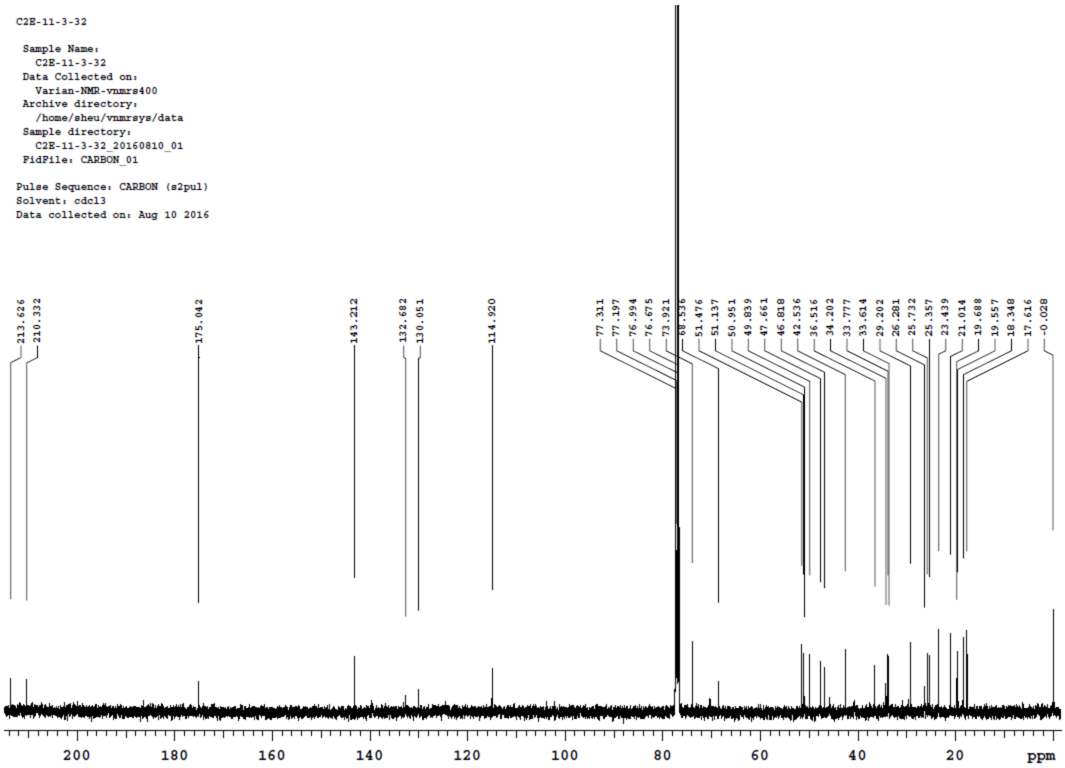


Figure S25: $^{13}$C NMR spectrum of **3** in $CDCl_3$ at 100 MHz

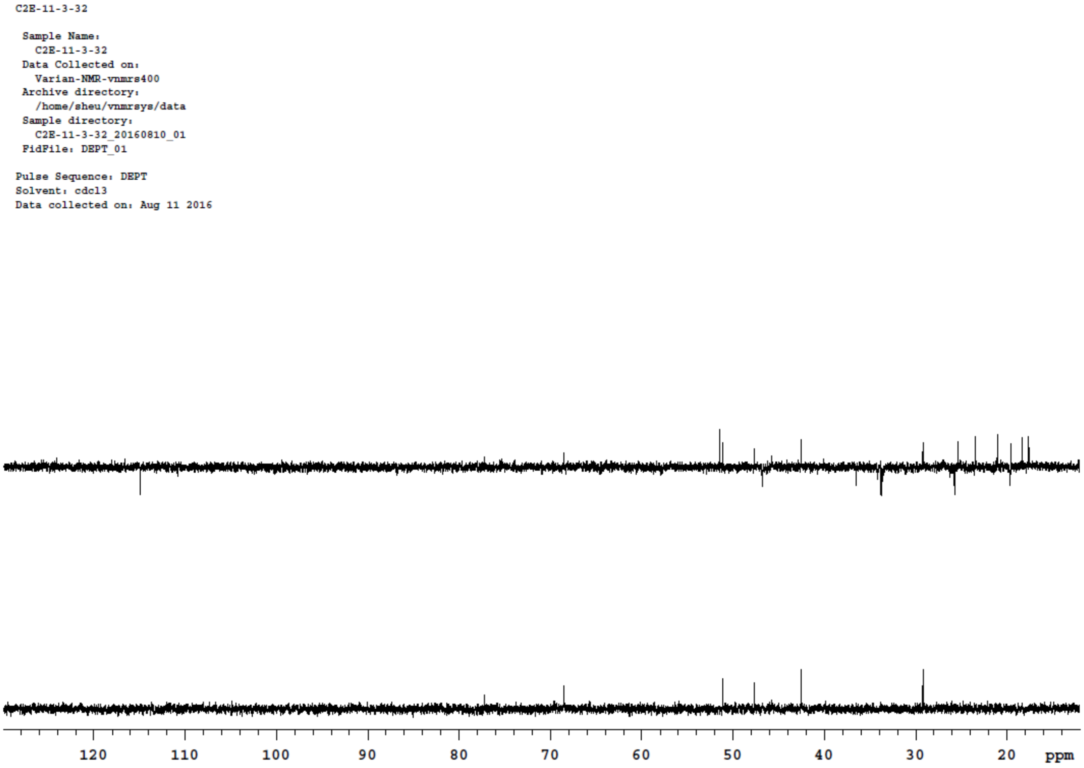


Figure S26: DEPT spectrum of **3**

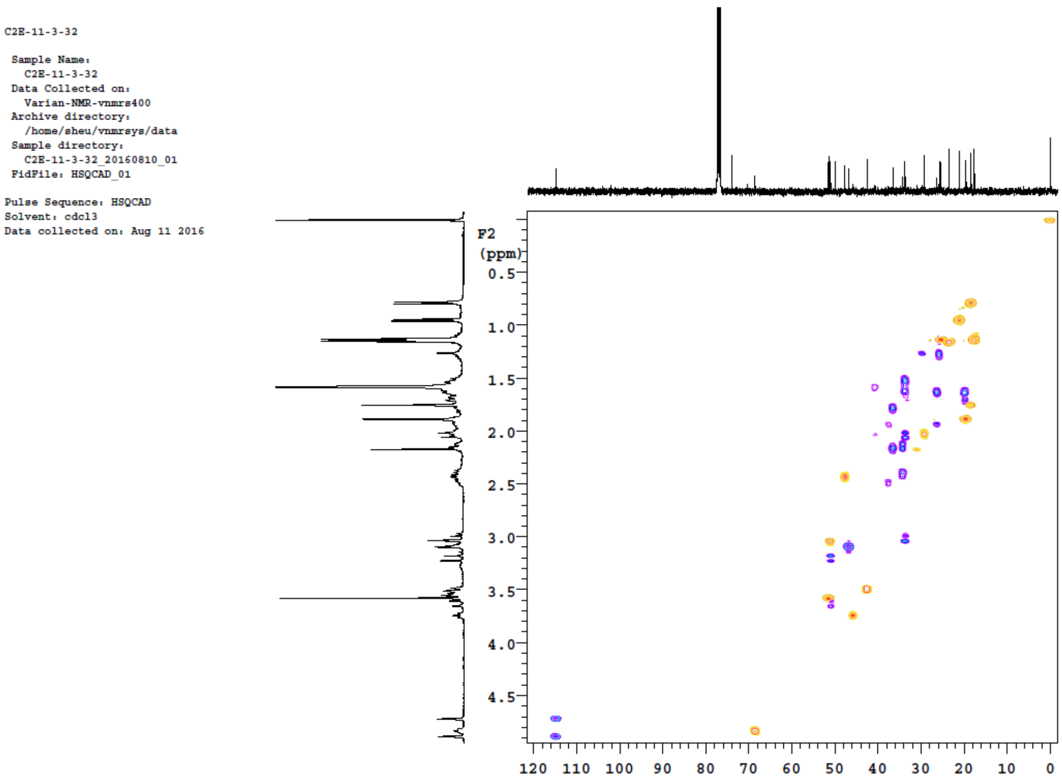


Figure S27: HSQC spectrum of **3**

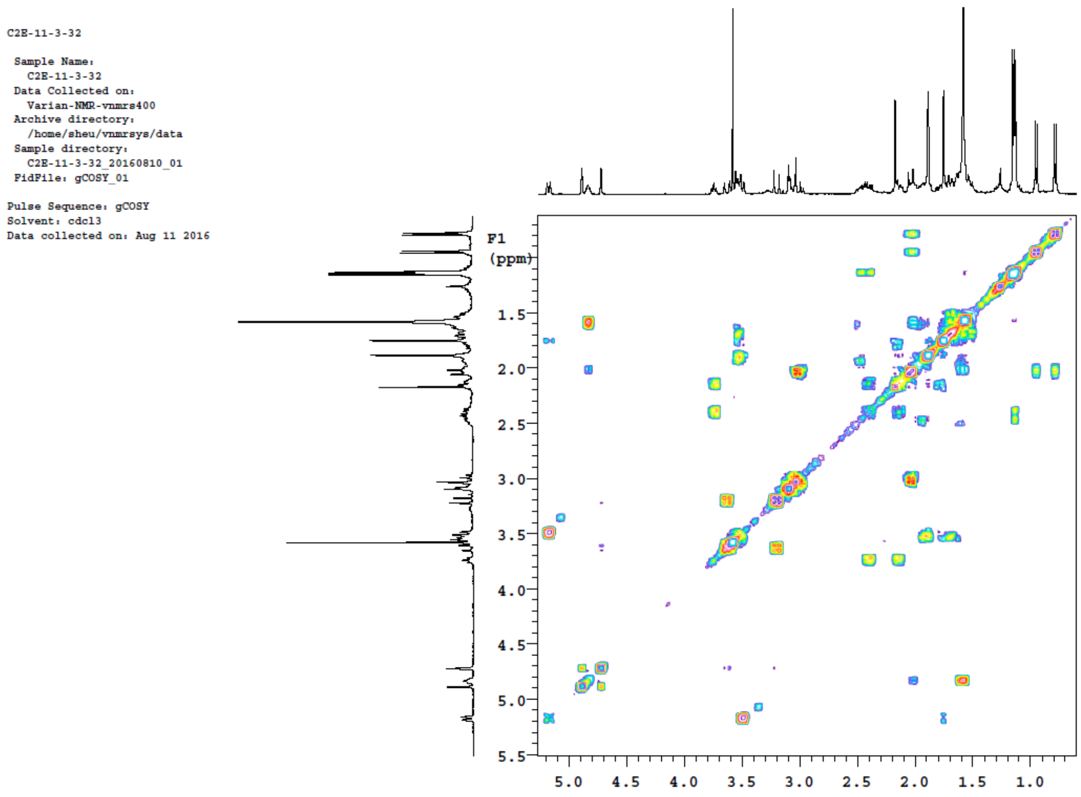


Figure S28: COSY spectrum of **3**

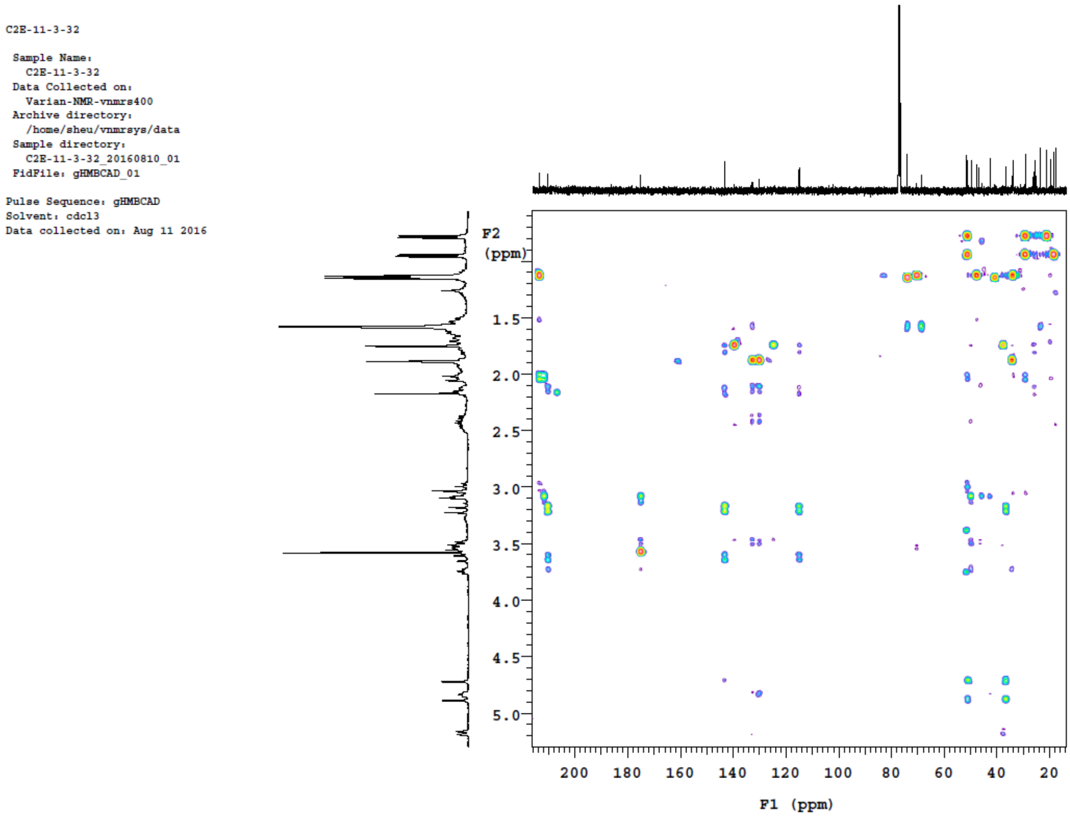


Figure S29: HMBC spectrum of **3**

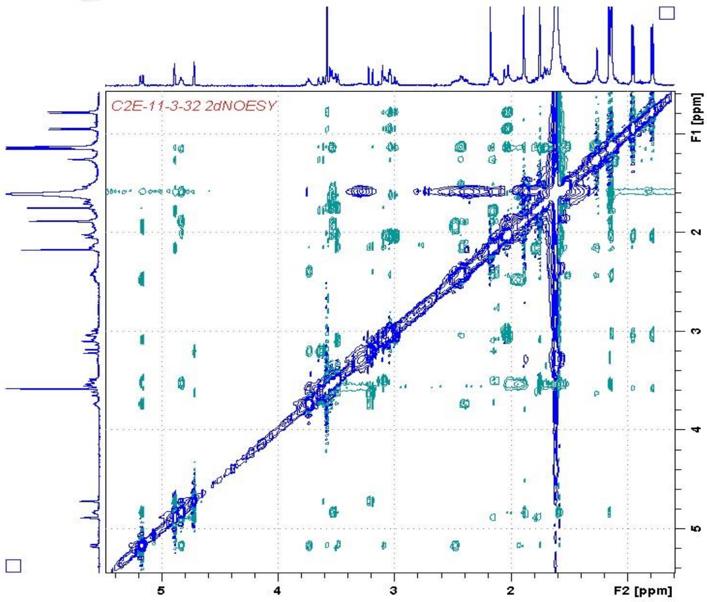


Figure S30: NOESY spectrum of **3**

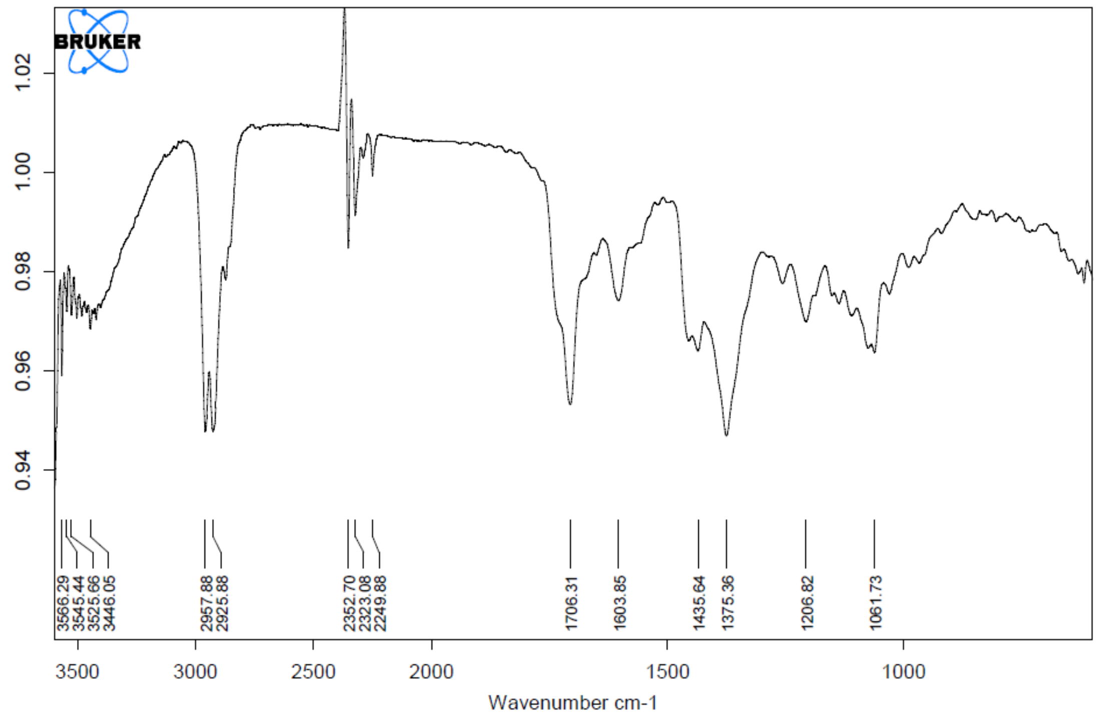


Figure S31: IR spectrum of **4**

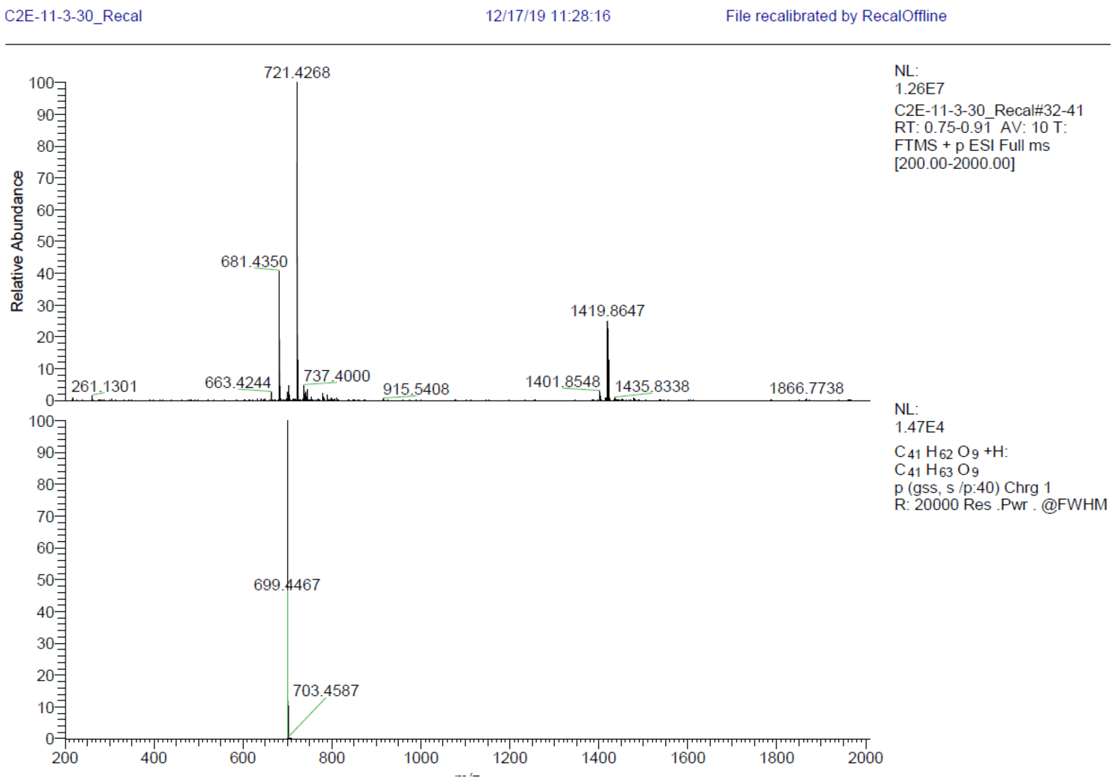


Figure S32: ESIMS spectrum of **4**

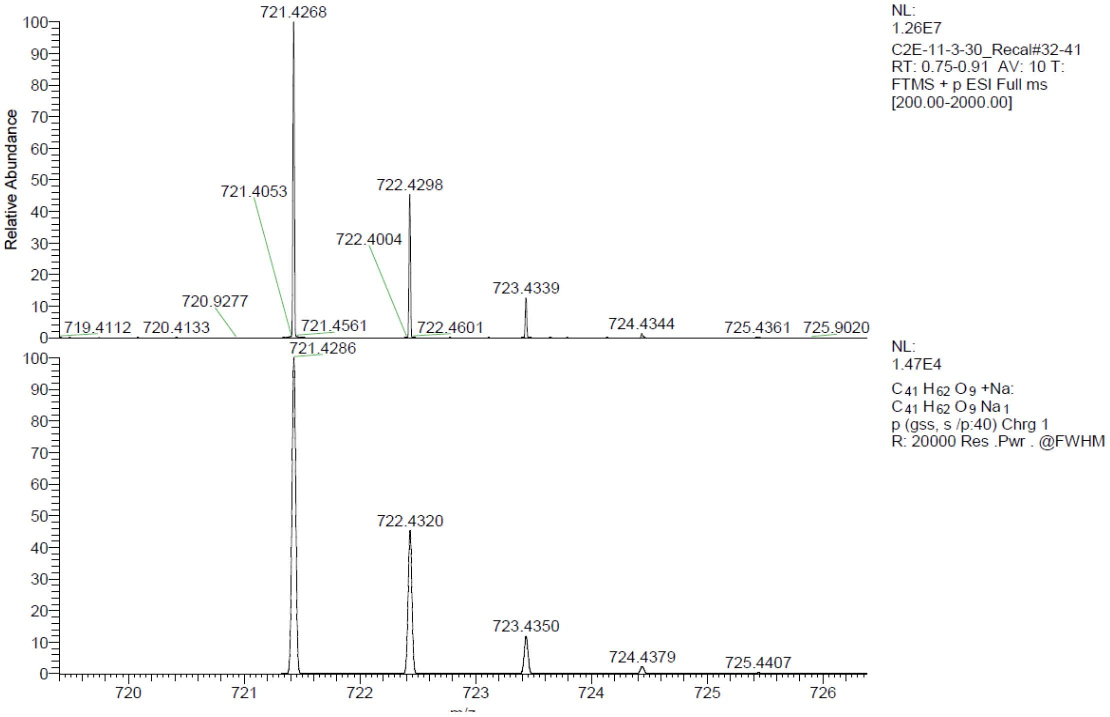


Figure S33: HRESIMS spectrum of **4**,

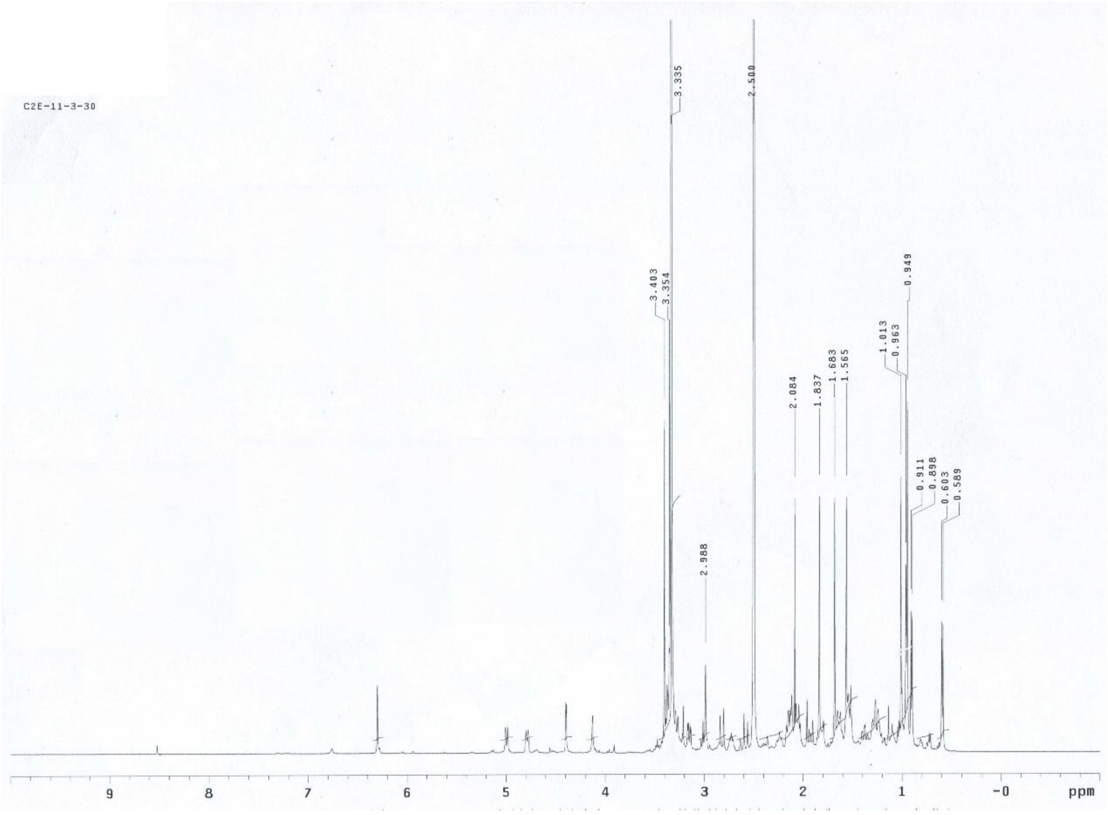


Figure S34: $^{1}$H NMR spectrum of **4** in DMSO-$d_6$ at 500 MHz

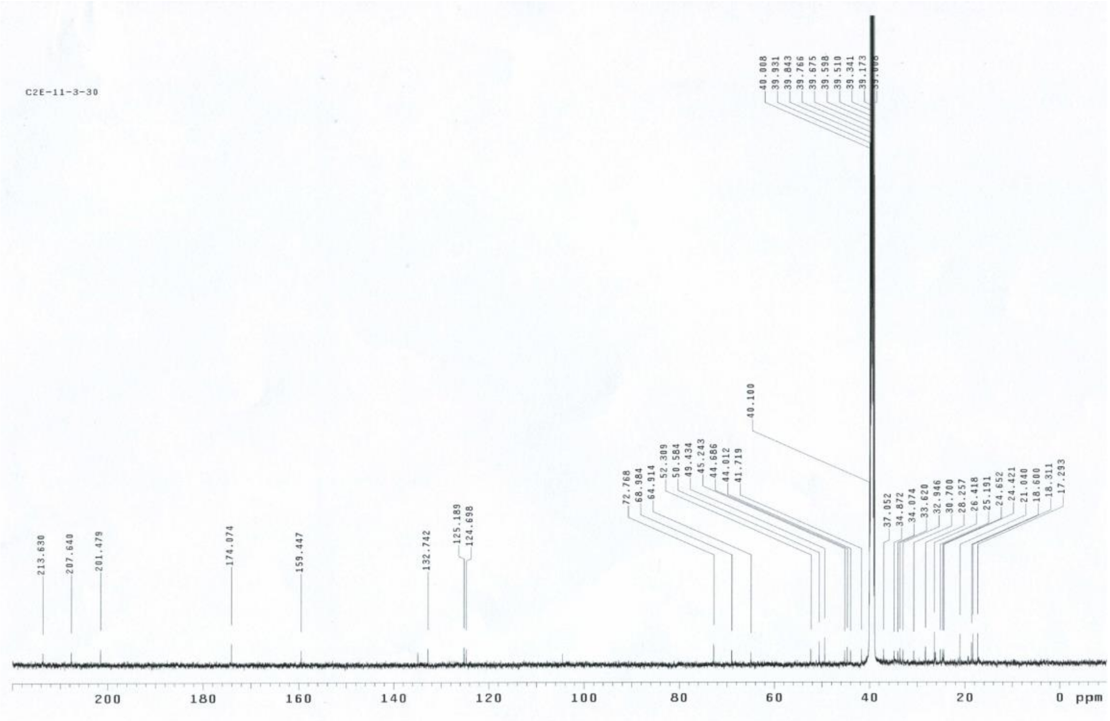

Figure S35: $^{13}$C NMR spectrum of **4** in DMSO-$d_6$ at 125 MHz

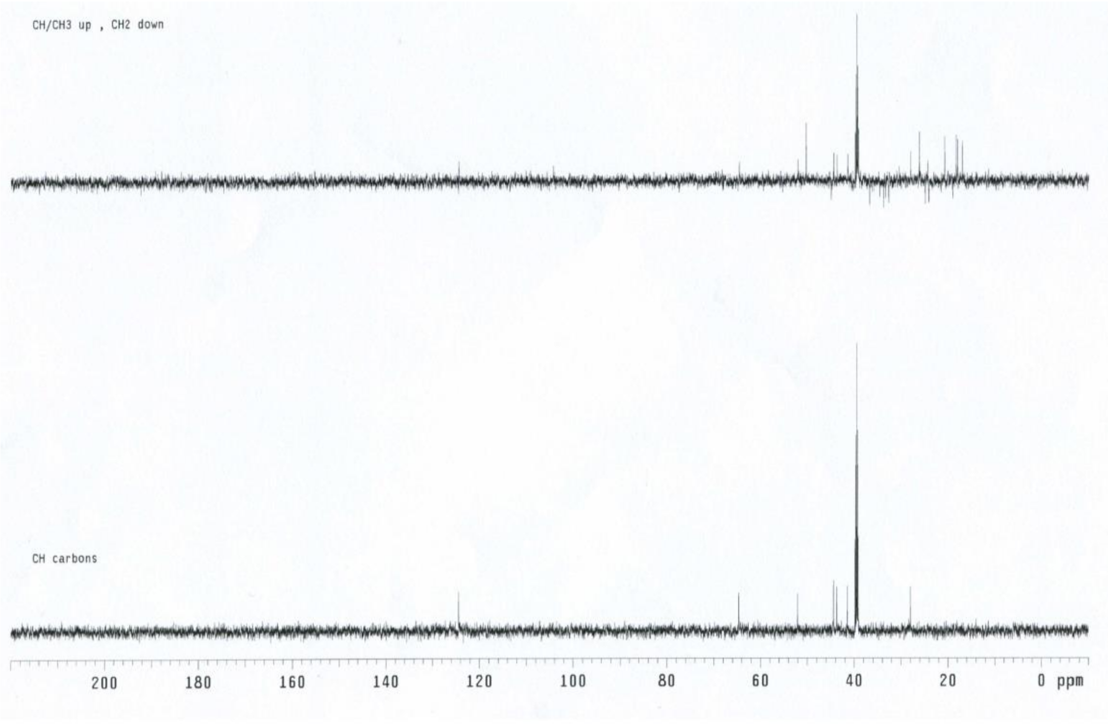


Figure S36: DEPT spectrum of **4**

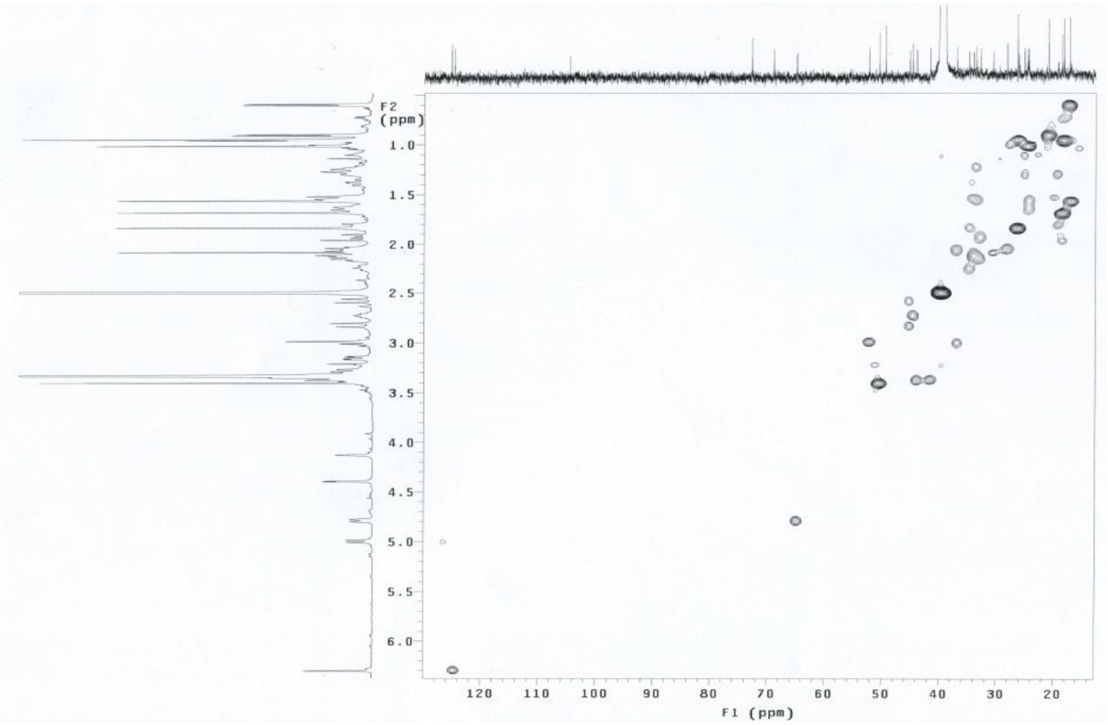


Figure S37: HSQC spectrum of **4**

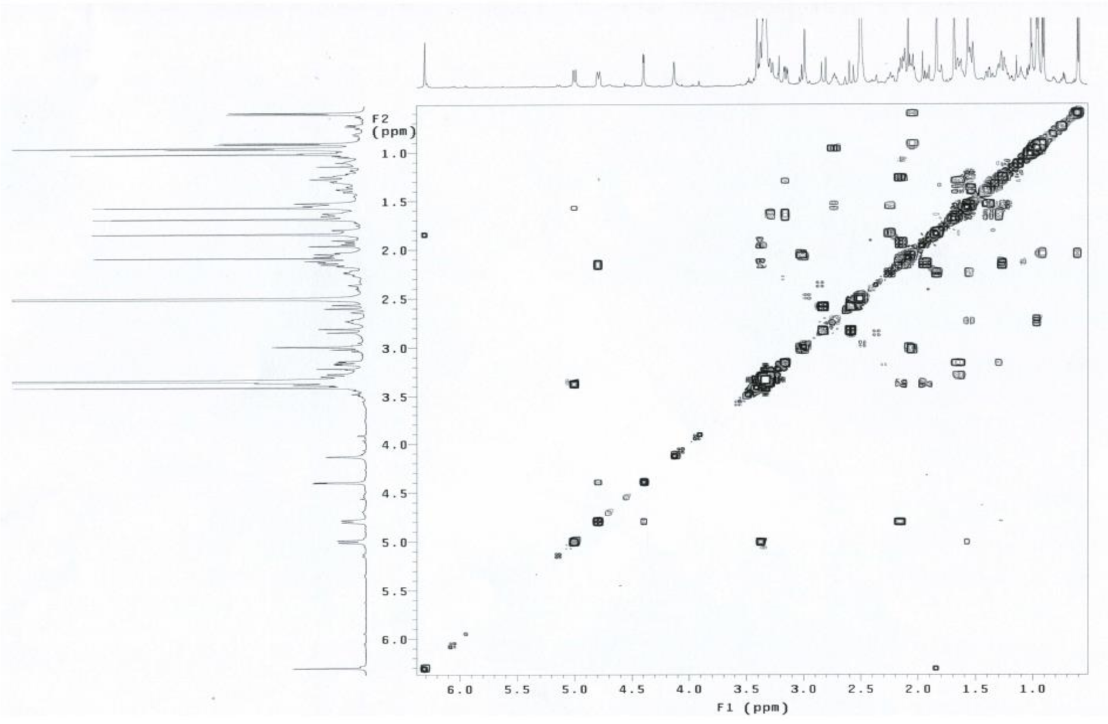


Figure S38: COSY spectrum of **4**

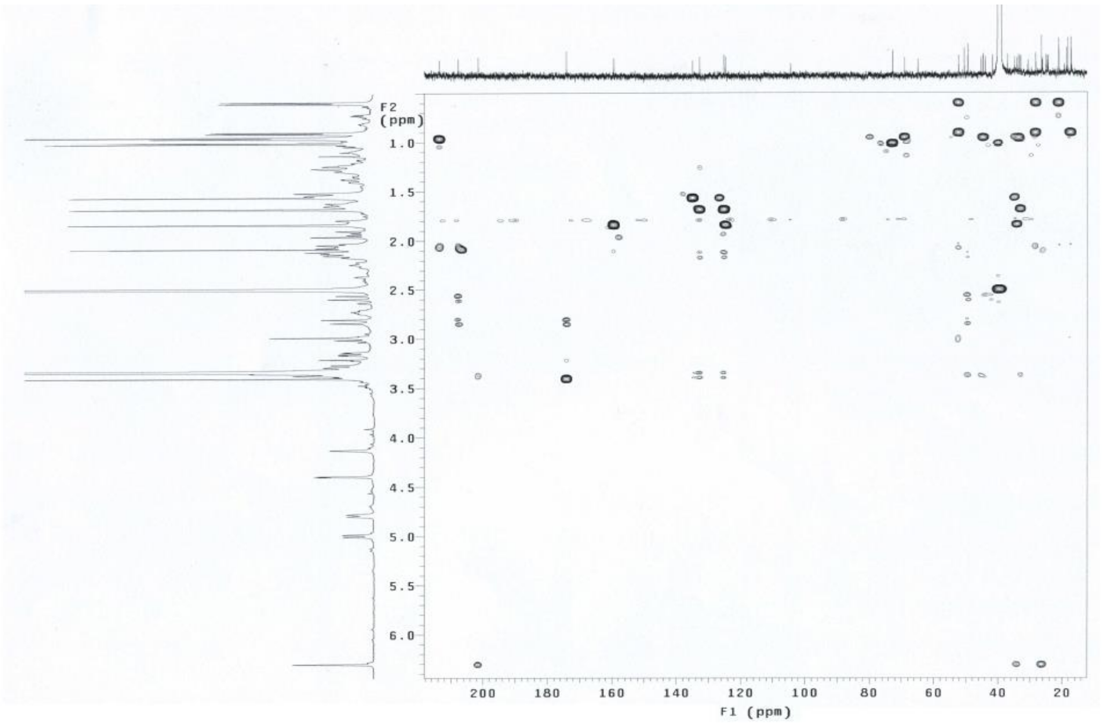


Figure S39: HMBC spectrum of **4**

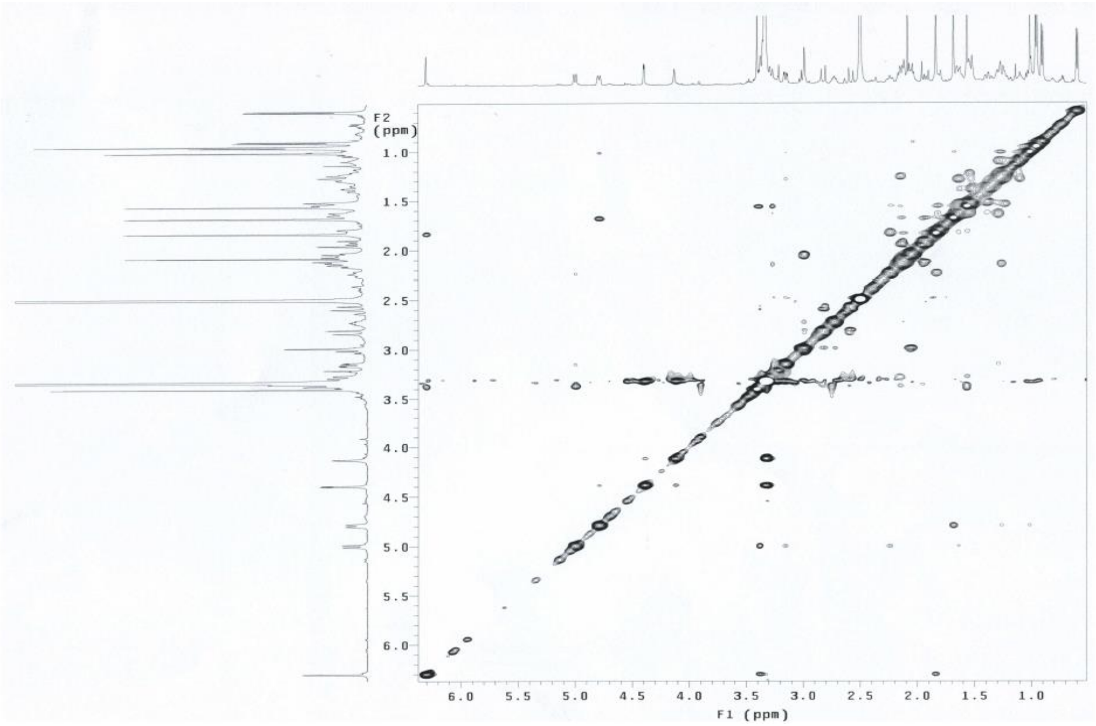


Figure S40: NOESY spectrum of **4**

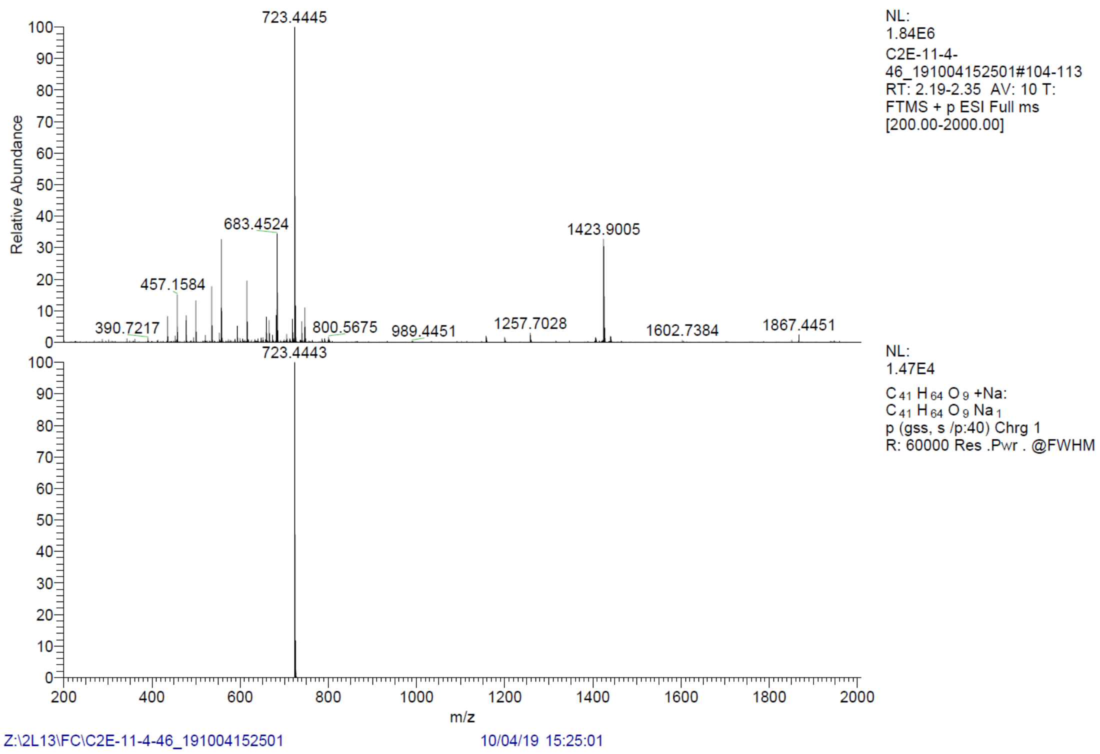


Figure S41: ESIMS spectrum of **5**

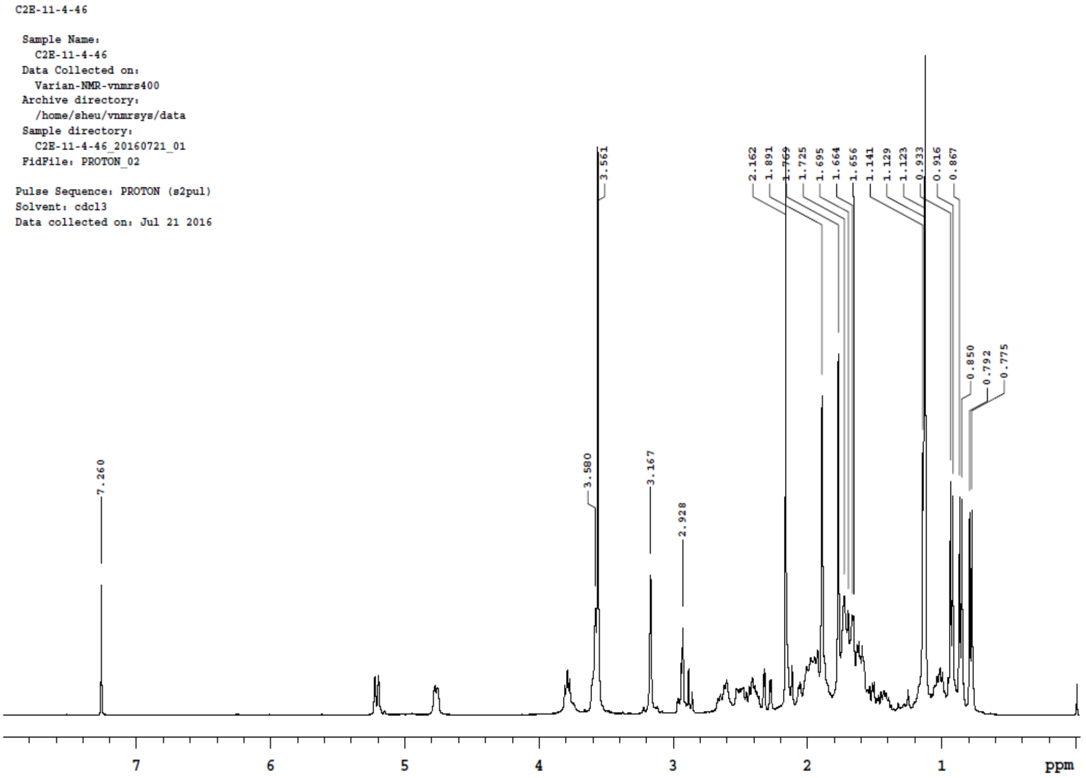


Figure S42: $^{1}$H NMR spectrum of **5** in $CDCl_3$ at 400 MHz

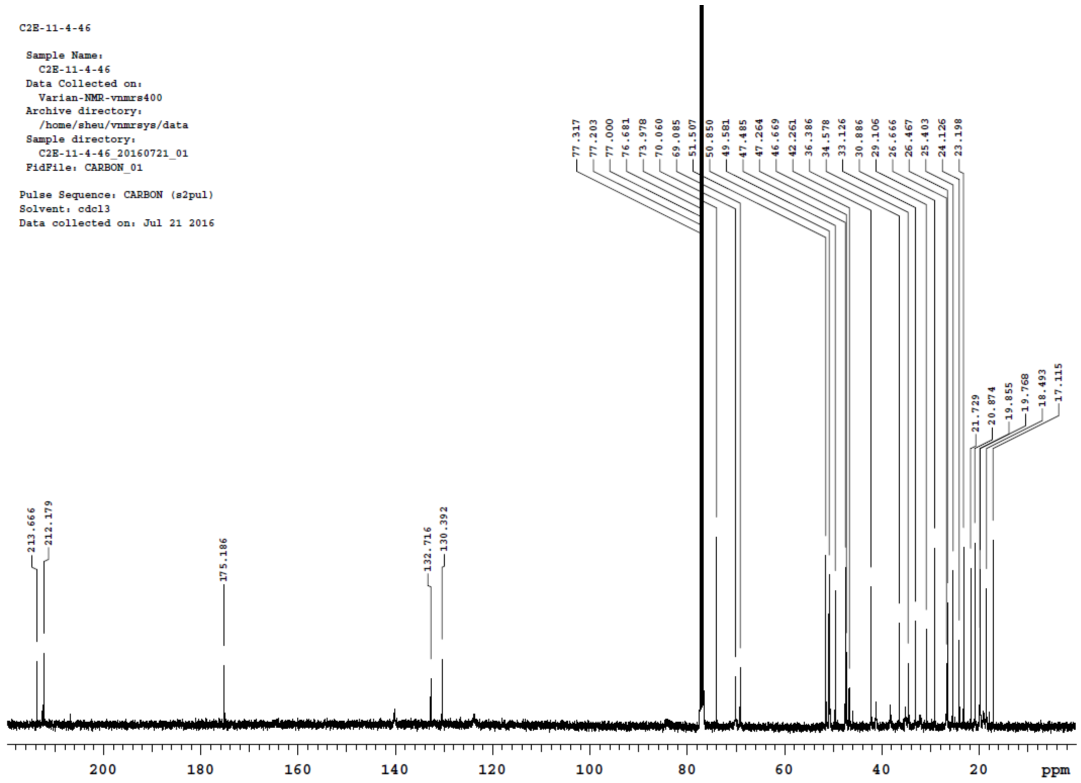


Figure S43: $^{13}$C NMR spectrum of **5** in $CDCl_3$ at 100 MHz

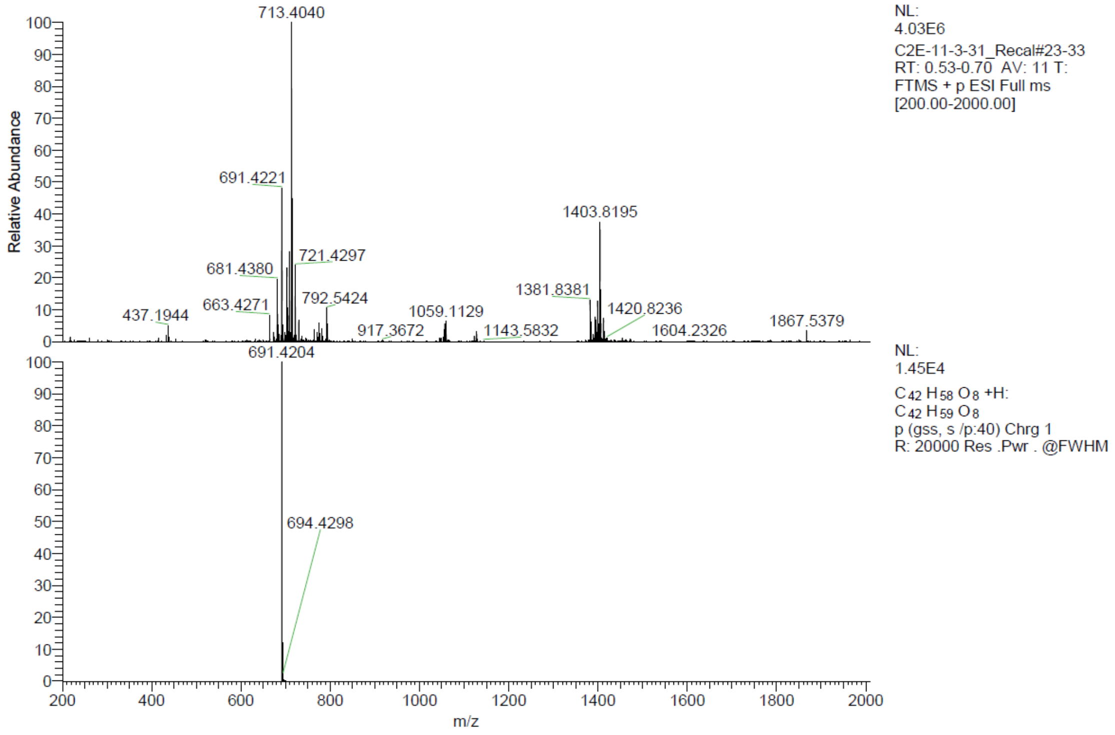


Figure S44: ESIMS spectrum of **6**

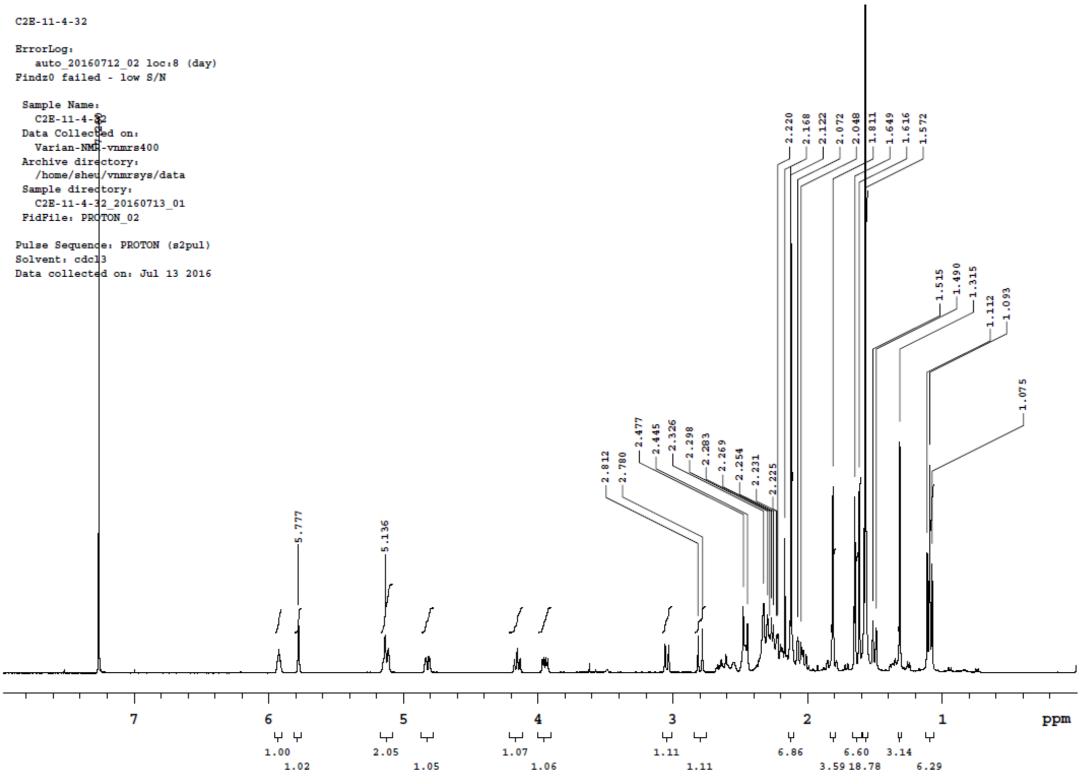


Figure S45: $^{1}$H NMR spectrum of **6** in $CDCl_3$ at 400 MHz

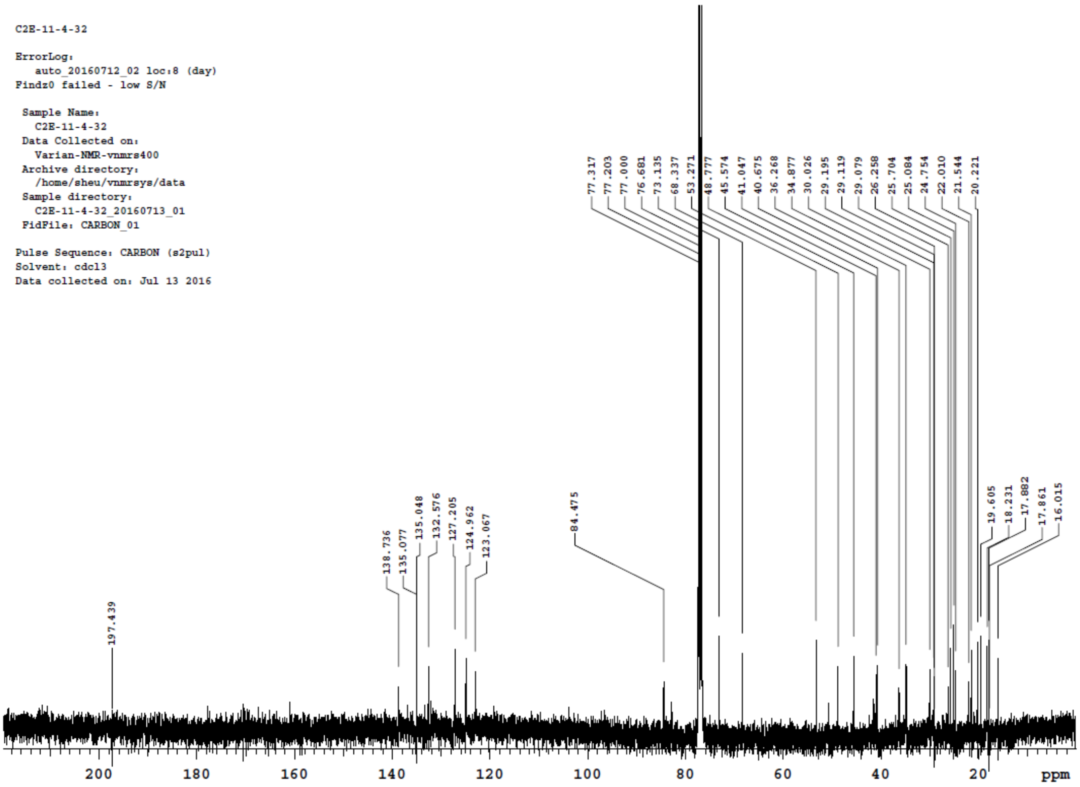


Figure S46: $^{13}$C NMR spectrum of **6** in $CDCl_3$ at 100 MHz

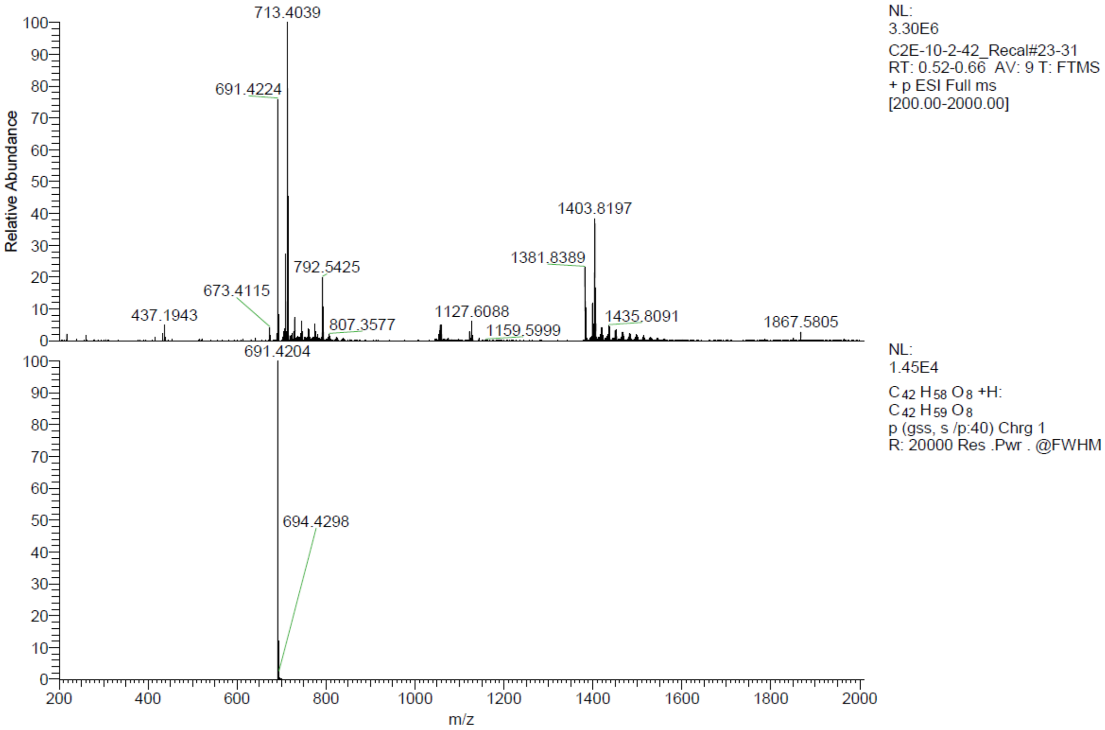


Figure S47: ESIMS spectrum of **7**

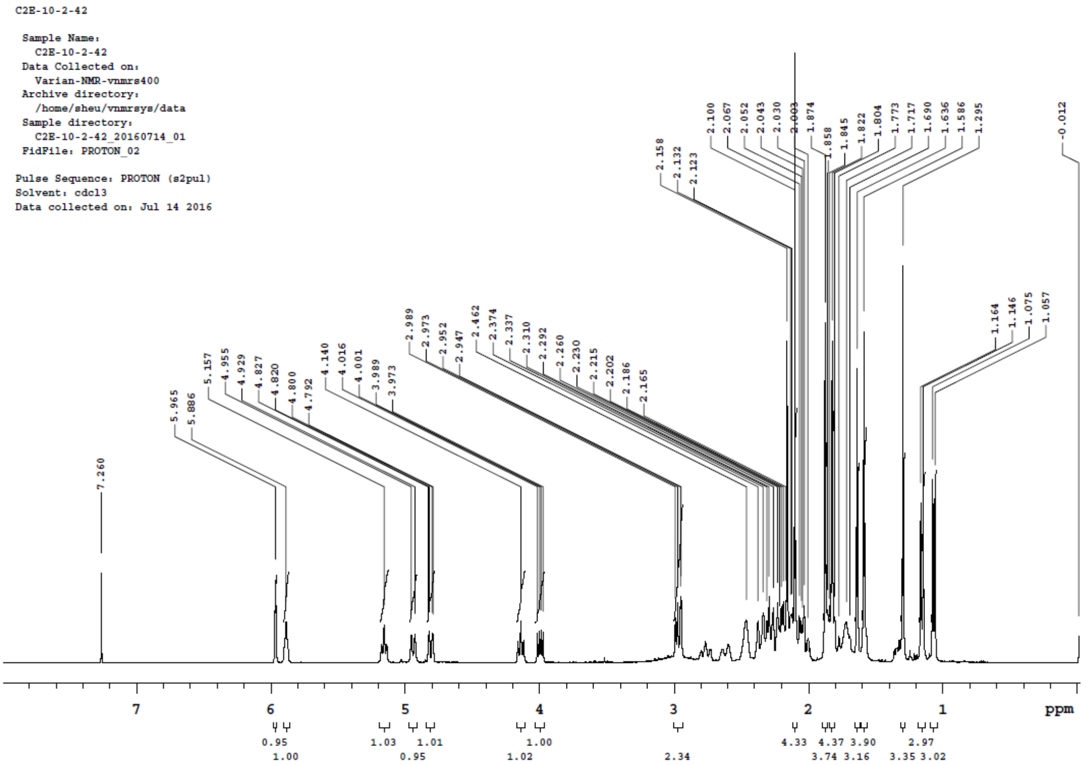


Figure S48: $^{1}H$ NMR spectrum of **7** in $CDCl_3$ at 400 MHz

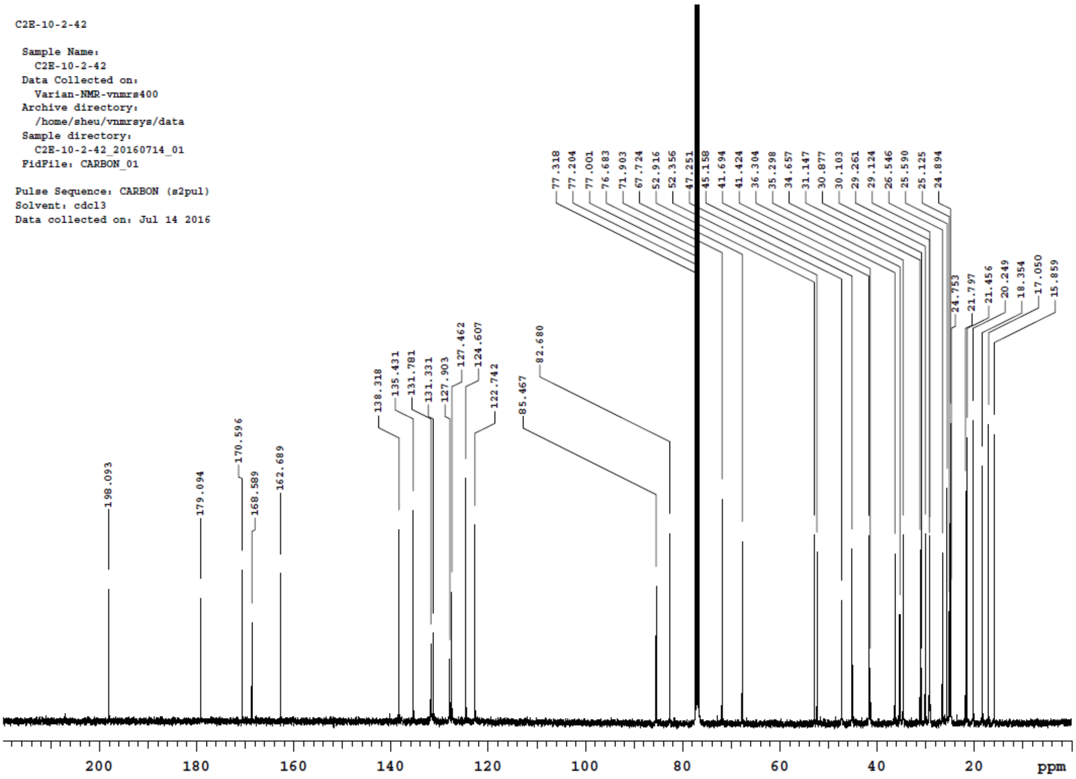


Figure S49: $^{13}$C NMR spectrum of **7** in $CDCl_3$ at 100 MHz

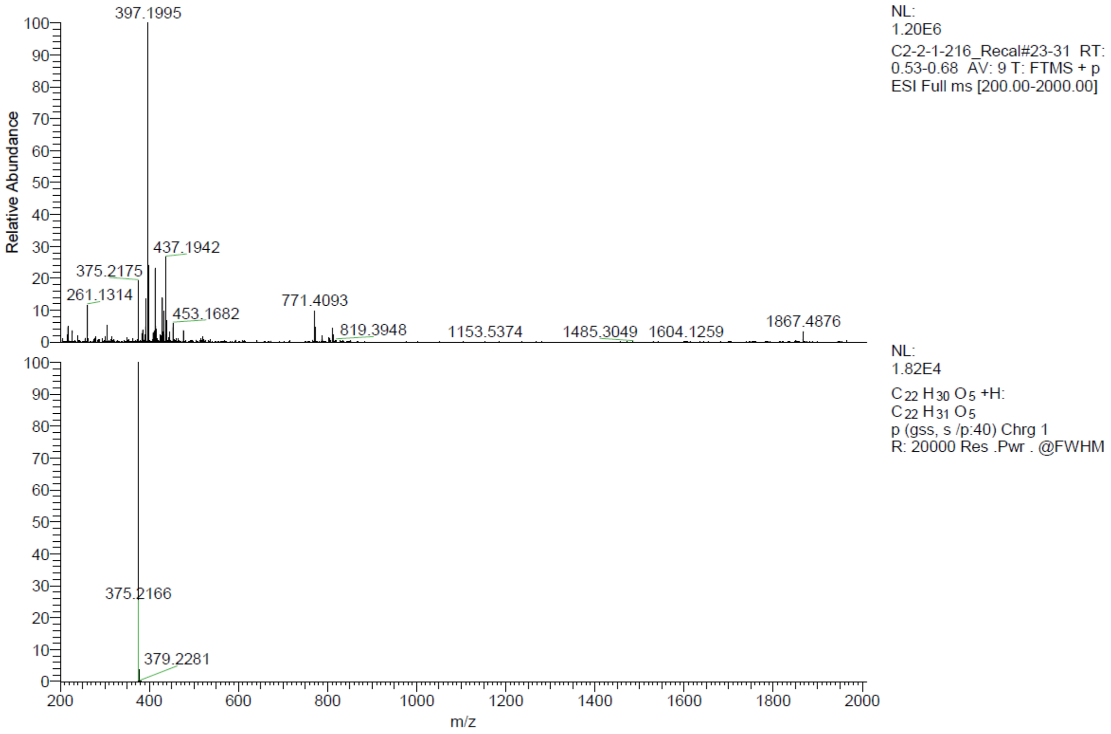


Figure S50: ESIMS spectrum of **8**

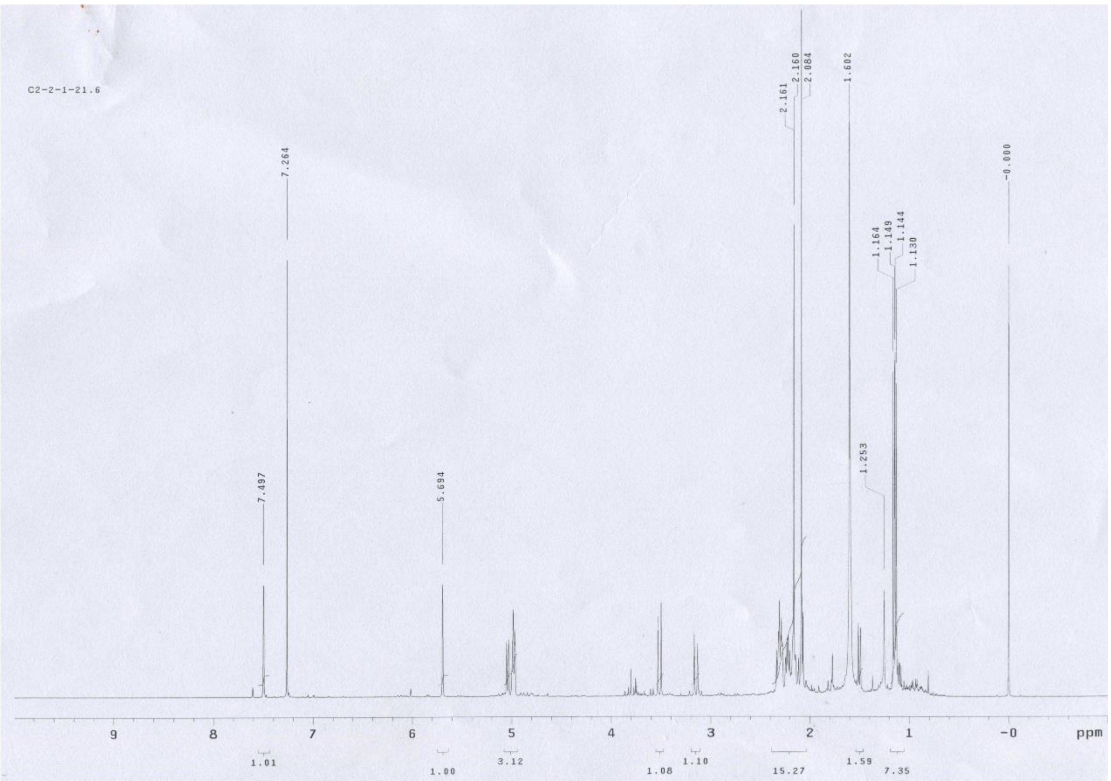


Figure S51: $^{1}H$ NMR spectrum of **8** in $CDCl_3$ at 500 MHz

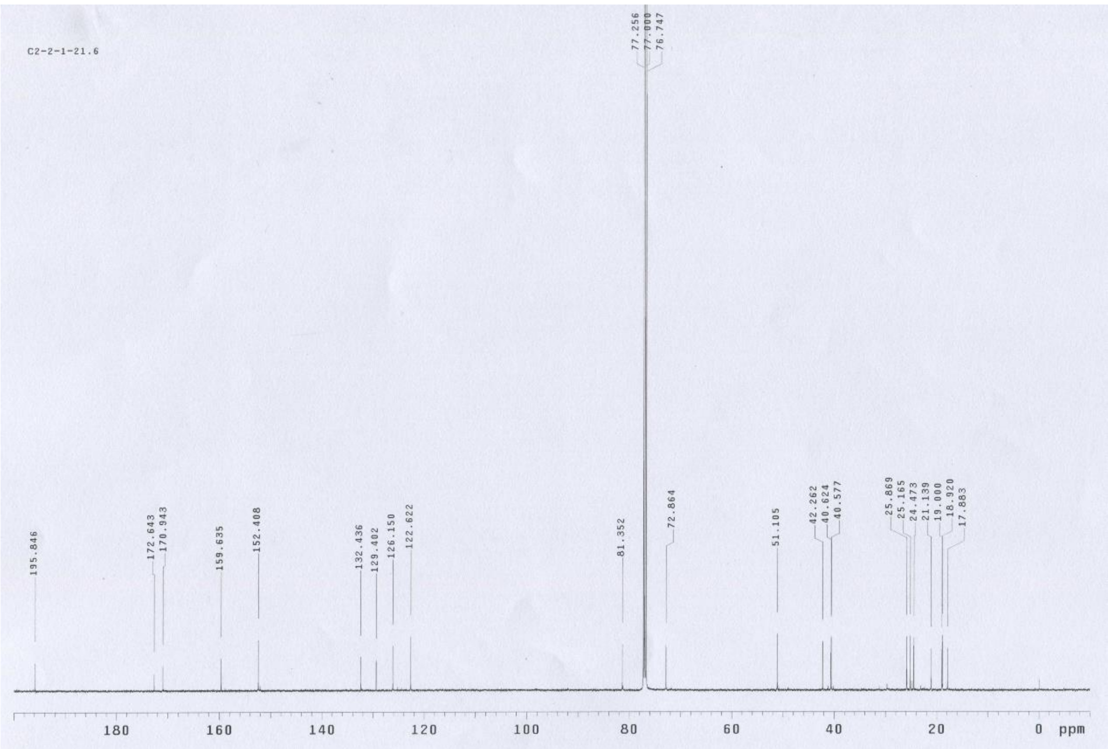


Figure S52: $^{13}C$ NMR spectrum of **8** in $CDCl_3$ at 125 MHz
